# Atom Exchange Versus Reconstruction: (Ge_*x*_As_4−*x*_)^*x*−^ (*x=*2, 3) as Building Blocks for the Supertetrahedral Zintl Cluster [Au_6_(Ge_3_As)(Ge_2_As_2_)_3_]^3−^

**DOI:** 10.1002/anie.202008108

**Published:** 2020-08-04

**Authors:** Fuxing Pan, Lukas Guggolz, Florian Weigend, Stefanie Dehnen

**Affiliations:** ^1^ Fachbereich Chemie and Wissenschaftliches Zentrum für Materialwissenschaften (WZMW) Philipps-Universität Marburg Hans-Meerwein-Straße 4 35043 Marburg Germany

**Keywords:** DFT calculations, gold, main group (semi)metals, X-ray diffraction, Zintl clusters

## Abstract

The Zintl anion (Ge_2_As_2_)^2−^ represents an isostructural and isoelectronic binary counterpart of yellow arsenic, yet without being studied with the same intensity so far. Upon introducing [(PPh_3_)AuMe] into the 1,2‐diaminoethane (en) solution of (Ge_2_As_2_)^2−^, the heterometallic cluster anion [Au_6_(Ge_3_As)(Ge_2_As_2_)_3_]^3−^ is obtained as its salt [K(crypt‐222)]_3_[Au_6_(Ge_3_As)(Ge_2_As_2_)_3_]⋅en⋅2 tol (**1**). The anion represents a rare example of a superpolyhedral Zintl cluster, and it comprises the largest number of Au atoms relative to main group (semi)metal atoms in such clusters. The overall supertetrahedral structure is based on a (non‐bonding) octahedron of six Au atoms that is face‐capped by four (Ge_*x*_As_4−*x*_)^*x*−^ (*x*=2, 3) units. The Au atoms bind to four main group atoms in a rectangular manner, and this way hold the four units together to form this unprecedented architecture. The presence of one (Ge_3_As)^3−^ unit besides three (Ge_2_As_2_)^2−^ units as a consequence of an exchange reaction in solution was verified by detailed quantum chemical (DFT) calculations, which ruled out all other compositions besides [Au_6_(Ge_3_As)(Ge_2_As_2_)_3_]^3−^. Reactions of the heavier homologues (Tt_2_Pn_2_)^2−^ (Tt=Sn, Pb; Pn=Sb, Bi) did not yield clusters corresponding to that in **1**, but dimers of ternary nine‐vertex clusters, {[AuTt_5_Pn_3_]_2_}^4−^ (in **2**–**4**; Tt/Pn=Sn/Sb, Sn/Bi, Pb/Sb), since the underlying *pseudo*‐tetrahedral units comprising heavier atoms do not tend to undergo the said exchange reactions as readily as (Ge_2_As_2_)^2−^, according to the DFT calculations.

## Graphical abstract

**The supertetrahedral cluster** [Au_6_(Ge_3_As)(Ge_2_As_2_)_3_]^3−^ is obtained from [(PPh_3_)AuMe] and the binary Zintl anion (Ge_2_As_2_)^2−^, which readily undergoes an in situ atom exchange in solution to form (GeAs^3^)^−^ and (Ge_3_As)^3−^. The latter is required to form the product, which comprises the largest number of Au atoms relative to main group (semi)metal atoms in such clusters.

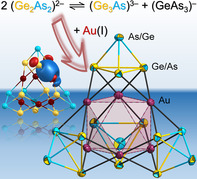

## Introduction

As a consequence of its metastable, yet controllable, character under ambient conditions, white phosphorus (P_4_) has been playing a vital role in phosphorus chemistry, given its multifaceted coordination modes and activation pathways.^1^ Its heavier congener yellow arsenic (As_4_) converts even more readily into the thermodynamic most stable gray allotrope, which complicates further use for coordination chemistry due to its layered structure. For this, the chemistry of As_4_ requires even more sophisticated strategies for its preparation, storage and controlled delivery to chemical reactions. These include the reversible coordination of As_4_ to transition metals^2^ or the encapsulation of As_4_ as guest in porous materials.^3^ Such techniques were developed and optimized during the recent past and enabled a fascinating and beautiful follow‐up chemistry towards transition metals and also main group compounds.^4^ The studies also allowed a facile access to the binary molecule AsP_3_ in condensed phase, which was previously studied in gas phase only.^5^

Another approach to work with (*pseudo*‐)tetrahedral units of main group semi‐metals is the isoelectronic replacement of some or all of the pnictogen atoms in Pn_4_ (Pn=P, As) with (formally) negatively charged atoms of group 13 or 14 elements. This is realized in salts of the corresponding Zintl anions, which show relatively high (thermal) stabilities and solubility in polar solvents. In the past decade, such binary *pseudo*‐tetrahedral anions were shown to be excellent starting materials to the formation of a large variety of heterometallic and intermetalloid Zintl clusters.^6^

With (Tt_2_Pn_2_)^2−^ anions (Tt=Ge, Sn, Pb; Pn=P, As, Sb, Bi), for instance, a variety of ternary clusters were obtained through interaction with d‐ and f‐block organometallic reagents.^7^, ^8^, ^9^, ^10^ In most of these cases, the anions underwent significant fragmentations and rearrangements—similar to reactions involving P_4_ and As_4_. For instance, (Sn_2_Sb_2_)^2−^ was drastically reorganized in a reaction with [LCu(NCMe)] (L=[{N(C_6_H_3_^*i*^Pr_2_‐2,6)C(Me)}_2_CH]^−^) to form a dimer of ternary 9‐vertex units, {[CuSn_5_Sb_3_]^2−^}_2_.7d The heteroatomic situation proved critical in monitoring and understanding of multimetallic cluster growth, as exemplified in comprehensive studies involving (Ge_2_As_2_)^2−^ during the stepwise formation of Ta/Ge/As clusters.^11^

The tendency to fragmentation is in sharp contrast to the behavior of homoatomic Zintl anions Tt_4_^4−^ (Si, Ge, Sn, Pb). Reactions of these species, all of which needed to be performed in liquid ammonia due to the corresponding salts’ poor solubility (in the absence of organic ligands),^12^ revealed the conservation of the tetrahedral units and their function as polyatomic ligands to Cu^+^, Au^+^ or Zn^2+^ ions.^13^, ^14^

**Figure 1:**
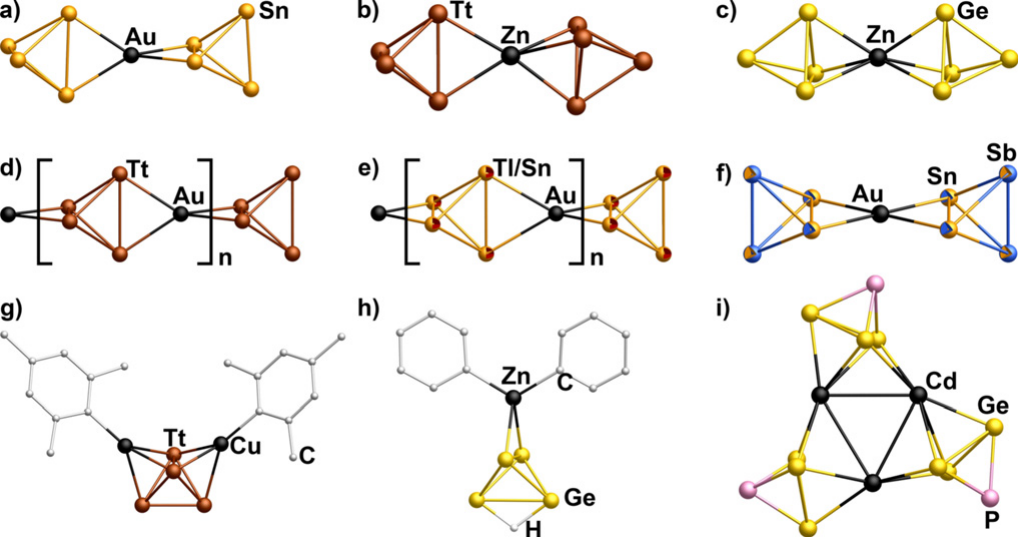
Overview of multimetallic complexes comprising *pseudo*‐tetrahedral Zintl anions as ligands of d^10^ metal atoms. a) [Au(η^2^‐Sn_4_)_2_]^7−^,13e b) [(η^2^‐Tt_4_)Zn(η^3^‐Tt_4_)]^6−^ (Tt=Ge,13c Sn14a), c) [(η^3^‐Ge_4_)Zn(η^3^‐Ge_4_)]^6−^,14b d) 1∞ [Au(η^2^:η^2^‐Tt_4_)]^3−^ (Tt=Sn, Pb),^15^ e) 1∞ [Au(η^2^:η^2^‐TlSn_3_)]^4−^,^16^ f) [Au(Sn_2_Sb_2_)]^3−^,^17^ g) [(MesCu)_2_(η^3^,η^3^‐Tt_4_)]^4−^ (Tt_4_=Si_4_, Ge_4_, Si_3.3_Ge_0.7_),13a, 13b, 13c h) [{η^2^‐(HGe_4_)}ZnPh_2_]^3−^,13d i) [Cd_3_(Ge_3_P)_3_]^3−^.7c Hydrogen atoms in organic groups are omitted for clarity.

Coinage metals and other d^10^ metals, in particular, have been witnessed of their strong abilities not only to activate P_4_ and As_4_, but also the isoelectronic group 14 tetrahedra mentioned above. The anionic clusters [(MesCu)_2_(η^3^,η^3^‐Tt_4_)]^4−^ (Tt_4_=Si_4_, Ge_4_, Si_3.3_Ge_0.7_) were among the first examples for successful solution chemistry with Tt_4_^4−^.13a, 13b, 13c Upon in situ formation, Sn_4_^4−^ tetrahedra were linked through a central gold atom to form the first binary Au/Sn polyanion [Au(η^2^‐Sn_4_)_2_]^7−^,13e which represents a cut‐out from chain‐like extensions of the type 1∞ [Au(η^2^:η^2^‐Tt_4_)]^3−^ (Tt_4_=Sn_4_, Pb_4_)^15^ or 1∞ [Au(η^2^:η^2^‐TlSn_3_)]^3−^ 16 that exist in solid phases. The ternary analog of the molecular species, [Au{η^2^‐(Sn_2_Sb_2_)}_2_]^3−^, was obtained in a relatively mild environment due to the reduced overall charge of the binary *pseudo*‐tetrahedron.^17^ Very recently, a ternary cluster anion, [Cd_3_(Ge_3_P)_3_]^3−^ was synthesized, comprising binary *pseudo*‐tetrahedral units.7c Besides proving (Ge_2_P_2_)^2−^ to be a suitable precursor for multinary cluster synthesis, the reaction product indicated an equilibrium of various (Ge_*x*_P_4−*x*_)^*x*−^ species to form in solution, which allowed for a heretofore unknown species (Ge_3_P)^3−^ to be available for the cluster synthesis. However, to the best of our knowledge, molecular architectures involving more than three anionic ligands or more than three metal atoms have not been known to date. An overview of structural motifs for the coordination of d^10^ metals by (*pseudo*‐)tetrahedral anions is provided in Figure 1.

## Results and Discussion

**Scheme 1:**
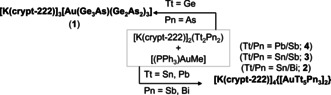
Summary of the reactions of salts of binary Zintl anions (Tt_2_Pn_2_)^2−^ with [Ph_3_PAuMe] in 1,2‐diaminoethane (en) yielding compounds **1**–**4**.

In this work, we describe the reaction of the binary Zintl anion [K(crypt‐222)]_2_(Ge_2_As_2_)11a, 11c with [(PPh_3_)AuMe] in en, yielding the supertetrahedral Zintl cluster [Au_6_(Ge_3_As)(Ge_2_As_2_)_3_]^3−^ as its [K(crypt‐222)]^+^ salt [K(crypt‐222)]_3_[Au_6_(Ge_3_As)(Ge_2_As_2_)_3_]⋅en⋅2 tol (**1**). Remarkably, reactions of the heavier homologous Zintl anions (Tt_2_Pn_2_)^2−^ (Tt=Sn, Pb; Pn=Sb, Bi) with the gold complex under identical reaction conditions yield yet another type of ternary heterometallic cluster. The anions of the products [K(crypt‐222)]_4_{[AuTt_5_Pn_3_]_2_}⋅*n* *Sol* (**2** for Tt/Pn=Sn/Bi, *n* *Sol*=2py; **3** for Tt/Pn=Sn/Sb, *n* *Sol*=2py; **4** for Tt/Pn=Pb/Sb, *n* *Sol*=3en) are isostructural to a corresponding copper cluster {[CuSn_5_Sb_3_]_2_}^4−^ that were reported only recently.7d We used quantum chemistry to shed light on the different reactivities of the binary anions used. Scheme 1 summarizes the reaction reported herein.

Compound **1** was characterized by means of single‐crystal X‐ray diffraction (SC‐XRD)^18^ and energy‐dispersive X‐ray spectroscopy (EDS, Figure S9). Electrospray‐ionization mass spectrometry (ESI‐MS) revealed fragmentation of the large cluster anion (M_w_=2360.01 g mol^−1^) under ESI‐MS conditions. Therefore, quantum chemical calculations were undertaken to confirm the number of Ge and As atoms in **1**. These studies also served to assign Ge and As atoms to their preferred positions within the cluster structure, and to analyze the electronic structure and the bonding situation.

**Figure 2:**
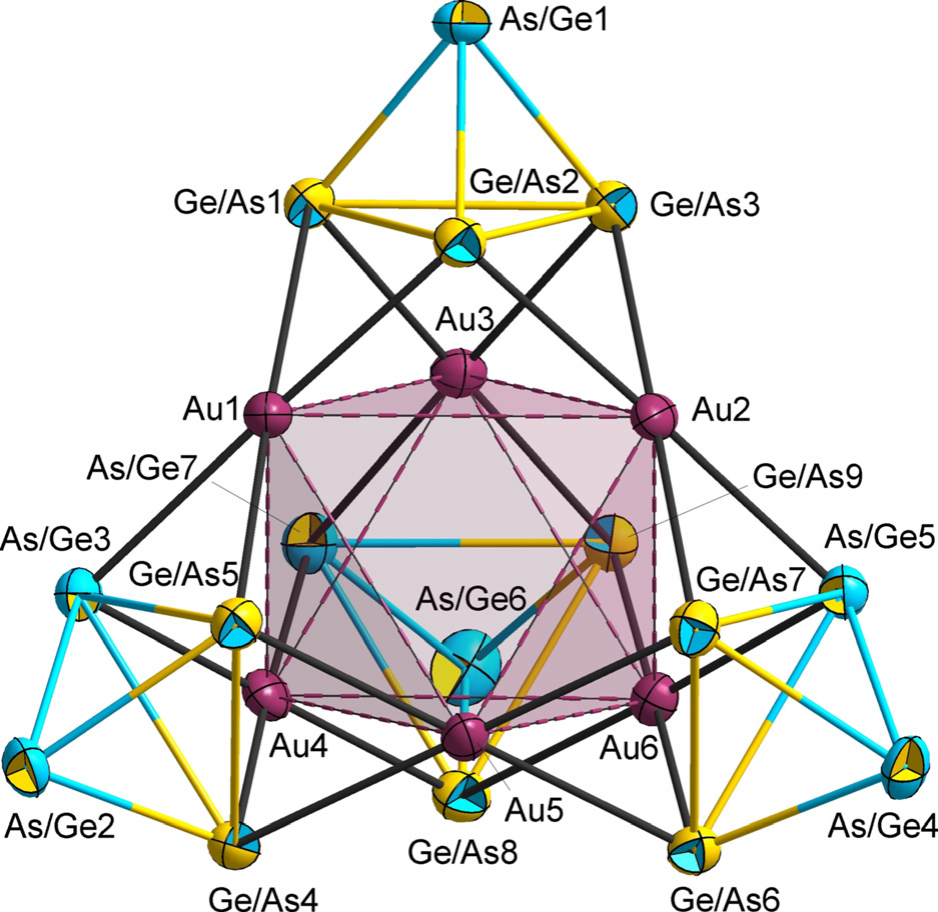
Molecular structure of the supertetrahedral cluster anion in **1**, with thermal ellipsoids drawn at 50 % probability. Since Ge and As atoms cannot be distinguished, the corresponding atom types are drawn as two‐colored atoms (yellow‐blue), with the more probable atom according to quantum chemical calculations being indicated by the dominant color (see text). Selected distances [Å] and angles [°]: As/Ge(apex)–Ge/As(basal) 2.4361(8)–2.4581(8), Ge/As(basal)–Ge(basal) 2.7879(8)–2.8279(7), Au–Ge/As 2.5369(5)–2.5662(6), Au⋅⋅⋅Au 3.516–3.615 (measured); Ge/As–Au–Ge/As 65.972(17)–67.420(17) and 111.075(18)–179.61(2).

According to SC‐XRD, the structure of the anion in **1** (Figure 2) is based on an octahedron of six Au atoms (Au⋅⋅⋅Au 3.516–3.615 Å), with half of the Au_3_ faces being capped by (Ge_*x*_As_4−*x*_)^*x*−^ (*x*=2, 3) *pseudo*‐tetrahedra in η^3^ fashion. The fourth apex of each of these units point away from the cluster center, hence giving the whole anion an overall supertetrahedral shape with (idealized) *T_d_* symmetry.

Since Ge and As atoms cannot be distinguished by means of common X‐ray diffraction experiments, the number and position of the main group atoms was determined in another way. First, we applied the *pseudo*‐element concept to rationalize the overall anionic charge of −3 that was unambiguously deduced from the number of three [K(crypt‐222)]^+^ cations per formula unit. Under the reasonable assumption of (formally) Au^+^ cations to be present (Au‐Ge/As 2.5369(5)–2.5661(6) Å), the total charge of the four *pseudo*‐tetrahedral units needs to sum up to −9. This would be achieved if three of the anionic main group units were (Ge_2_As_2_)^2−^ while one of them was (Ge_3_As)^3−^. Alternatively, the three lower‐charge units could be protonated (Ge_3_AsH)^2−^. Several methods were applied to clarify, which of these options would be the correct one. First, we checked the elemental composition by means of EDS. This revealed atom% values of K:Au:Ge:As to be 6.8 %:26.3 %:36.9 %:30.0 % (theoretical values calculated for K_3_Au_6_Ge_9_As_7_: 12.0 %:24.0 %:36.0 %:28.0 %). As a deviation of the K amount is rather common for Zintl cluster compounds, we also compared the Au:Ge:As percentages alone, which are 28.2 %:39.6 %:32.2 % according to the EDS measurement, hence in very good agreement with the theoretical values of Au_6_Ge_9_As_7_ (27.3 %:40.9 %:31.8 %). For comparison, the theoretical values for a protonated cluster K_3_Au_6_Ge_12_As_4_H_3_ would be K:Au:Ge:As=12.0 %:24.0 %:48.0 %:16.0 % and Au:Ge:As=27.3 %:54.5 %:18:2 %, which clearly is too far apart from the measured data.

To further corroborate our assumed composition, and to clarify the distribution of the Ge and As atoms on the atomic positions, we additionally applied DFT calculations^19^, ^20^, ^21^, ^22^, ^23^, ^24^ along with first order perturbation theory in the nuclear charge.^25^ These led to the atom assignment that is indicated by the dominant color of the two‐colored displacement ellipsoids drawn in Figure 2 above. According to these calculations, the Ge atoms are clearly preferred as coordinating atoms: all *pseudo*‐tetrahedral units are orientated such that one As atom points outwards and is not involved in the coordination. Hence, the (Ge_3_As)^3−^ unit binds with all three Ge atoms, and the (Ge_2_As_2_)^2−^ ligands involve two Ge atoms and one As atom each. In a recent comprehensive study of binary *pseudo*‐tetrahedral anions,^26^ we inspected the atomic orbitals of all different elemental combinations. The Ge–Ge bonds contribute predominantly to the HOMOs of these anions, so their involvement in the coordination is obvious. Still, the relative orientation of the 4‐vertex units allows for some isomeric architectures. The global minimum structure (illustrated in Figure 2 by the dominant color of the two‐colored ellipsoids) exists in two enantiomeric forms, with the second enantiomer resulting from an exchange of As/Ge7 and Ge/As9. In both enantiomers, one Au atom is coordinated by 4 Ge atoms, one Au atom exhibits a 2:2 ratio of Ge:As neighbors, and four Au atoms exhibit a 3:1 ratio of Ge:As neighbors, which is the best approximation of an equal coordination environment of all six atoms. In the second possible pair of isomers, given the precondition that four As atoms are not involved in the coordination of Au atoms (+7 kJ mol^−1^), 4:0, 3:1, and 2:2 ratios of coordinating atom types are realized for two Au atoms each, which is a more significant deviation from equality. As can be seen from the energy difference between these isomers, this is a slight, yet noticeable effect, as the total number of coordinating Ge and As atoms are the same in these species.

**Figure 3:**
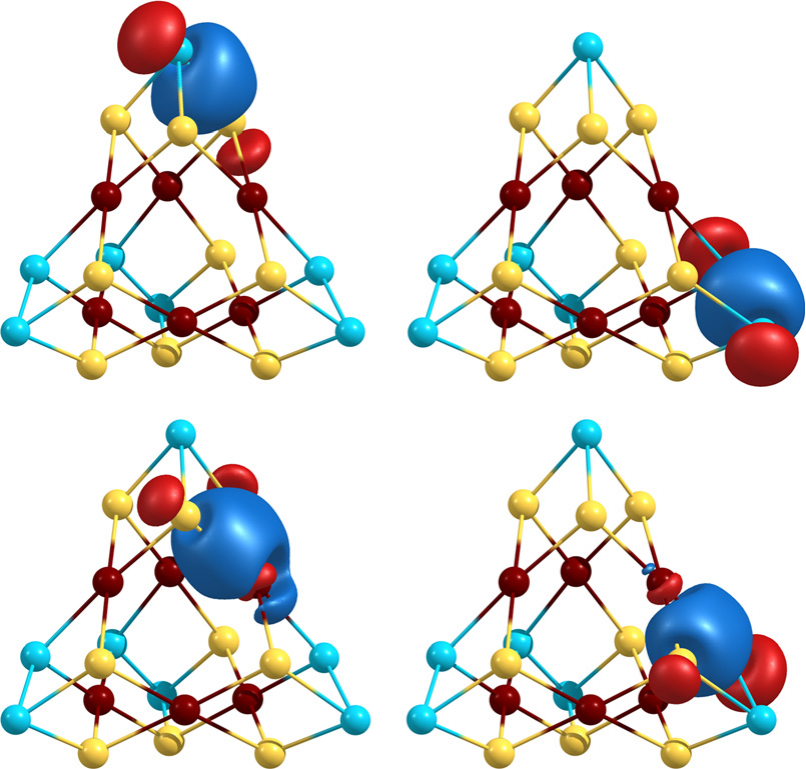
Images of representative localized molecular orbitals (LMOs) of the calculated global minimum structure of the [As_6_(Ge_3_As)(Ge_2_As_2_)_3_]^3−^ anion in **1**. The color code and the orientation are the same as in Figure 2. Contours are drawn at 0.048 a.u., for details see the text.

For elucidation of the bonding situation, a localization procedure was applied to the 70 valence orbitals, see Figure 3.

One obtains for each of the four outer As atoms three two‐center bonds to its three As or Ge neighbors—polarized in case of Ge (Figure 3, top left) and non‐polarized in case of As (Figure 3, top right). Each of the six Au atoms form two three‐center bonds with two atoms from the same 4‐vertex unit each. The three‐center bond contains similar contributions from the three atoms in case of the AuGe_2_ interaction (Figure 3, bottom left), while it is polarized towards As in case of the AuGeAs interaction (Figure 3, bottom right). The remaining 46 localized orbitals represent five d‐orbitals for each of the Au atoms and one lone pair for each of the As or Ge atoms. We note that there is no evidence for significant Au⋅⋅⋅Au interactions.

Another issue that required clarification is the observation of the *pseudo*‐tetrahedral anion (Ge_3_As)^3−^ that has not yet been isolated in a salt on its own. While it is a common observation to find fragmentation and rearrangement of the *pseudo*‐tetrahedral binary Zintl anions to larger moieties in the presence of transition metal atoms or ions (see also the reactions leading to compounds **2**–**4**), a reorganization of the 4‐vertex units themselves has only been detected by mass spectrometry in some cases, and in the case of the lightest known binary species, Ge_2_P_2_, a corresponding unit was included in the ternary complex [Cd_3_(Ge_3_P)_3_]^3−^ mentioned above.7c We assume that such species are formed in exchange reactions according to Equation (1), representing equilibria of different binary species in solution [note that Eq. (1) also indicates the formation of the homoatomic analogs, as the extreme of such exchange reactions (in grey); these species were not detected so far, but may occur as transient species, as we find red phosphorous in solutions involving Pn=P]. Very obviously, the elemental combinations involved in the binary anions seem to be critical to the relative abundance, or the occurrence of such equilibria at all, instead of further fragmentation.

(1) $$2\;\left(\mathrm{Tt}{}_{2}\mathrm{Pn}{}_{2}\right){}^{2-}\vec{\leftarrow }\left(\mathrm{Tt}{}_{3}\mathrm{Pn}\right){}^{3-}+\left(\mathrm{TtPn}{}_{3}\right){}^{-}\;\left[\vec{\leftarrow }\left(\mathrm{Tt}{}_{4}\right){}^{4-}+\mathrm{Pn}{}_{4}\right]{}^{\neq }\;$$

Evidence for this reaction to actually take place is given by ESI mass spectra of the reaction solution, in which we identified all three species with Ge:As ratios of 2:2, 3:1, and 1:3 to coexist (Figures S14–S17).

**Figure 4:**
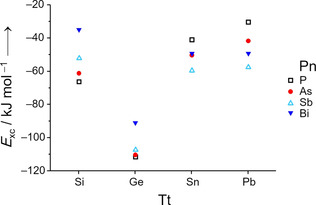
Illustration of the reaction energies for the exchange reactions, *E*_XR_, corresponding to the first step of Equation (1). Tt indicates the involved tetrel atom, Pn indicates the involved pnictogen atom.

To comprehend this observation and the fact that such exchanged *pseudo*‐tetrahedra were only observed for the anion in **1** and for [Cd_3_(Ge_3_P)_3_]^3−[7c]^ so far, we calculated reaction energies *E*_XR_ for the first step of this equilibrium, considering all—known or hypothetical—elemental combinations of Tt and Pn from periods 3–6 (Figure 4 and Table S8).

As discussed recently, the ratio of the atomic radii seems to play a role for the (relative) stabilities of binary *pseudo*‐tetrahedral anions.^26^ Hence we analyzed the results obtained for *E*_XR_ in this regard, and found that within a series of a given Tt, the course of the *E*_XR_ values indeed can be reproduced this way: obviously, for smaller differences of the covalent radii, the exchange reactions become more exoenergetic (Table S9). Hence anions with a 3:1 or 1:3 ratio of the involved atom types are stabilized more efficiently in comparison to the anions with a 2:2 atomic composition, if the sizes of the involved atoms are more similar; we attribute this to the fact that the 3:1 and 1:3 anions can be understood as triangles of one atom type with a single atom of the other type capping it, for which the size difference matters more than for two Tt−Tt or Pn−Pn “dumbbells” forming the 2:2 anions. Although these geometric consideration alone do not explain the particular situation of the Ge species (Ge_*x*_Pn_4−*x*_)^*x*−^, we are delighted to find the exchange reactions involving Ge to be distinctly more preferable than calculated for any other Tt/Pn combination—with the (Ge_2_P_2_)^2−^ anion being only slightly more prone to follow Equation (1) than (Ge_2_As_2_)^2−^. This finding is in excellent agreement with our experimental observations, in which only the exchange reaction products of (Ge_2_P_2_)^2−^ and (Ge_2_As_2_)^2−^ seem to form in sufficiently large amounts in solution to be available as ligands for transition metal atoms. According to the calculations, we suggest that the elemental combination Ge/Sb should show a similar behavior—yet, the underlying anion has not yet been explored to date.

**Figure 5:**
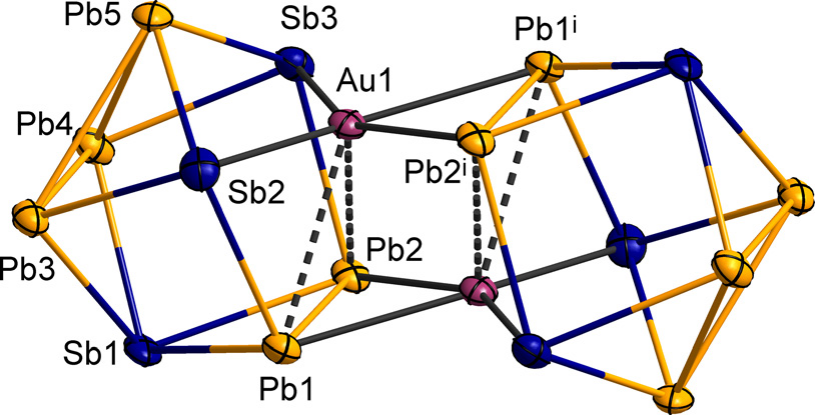
Molecular structure of the dimeric cluster anion in **4**, as an example of the isostructural cluster anions observed in compounds **2**–**4**. Thermal ellipsoids are drawn at 50 % probability. Selected distances [Å] and angles [°]: Pb1–Pb2 3.3114(7), Pb3–Pb4 3.1444(7), Pb(3,4)–Pb5 3.2268(7)–3.2285(7) Sb1–Pb 3.0101(9)–3.0703(13), Sb(2,3)–Pb 2.9571(9)–3.0035(10), Au1–Pb(1^i^,2^i^) 2.8671(7)–2.8951(12), Au1⋅⋅⋅Pb(1,2) 3.2236(6)–3.2418(8), Au–Sb 2.7293(8)–2.7475(10), Au⋅⋅⋅Au^i^ 2.8957(8); Pb1⋅⋅⋅Au1⋅⋅⋅Pb2 61.616(18), Pb1^i^–Au1–Pb2^i^ 70.15(2), Pb(1^i^,2^i^)–Au⋅⋅⋅Pb(2,1) 87.75(2), 88.57(2), Pb(1^i^,2^i^)–Au⋅⋅⋅Pb(1,2) 123.819(17), 123.812(16), Sb–Au–Sb 117.47(3), Sb(2,3)–Au–Pb(2^i^,1^i^) 85.52(3), 85.823(19), Sb(2,3)–Au–Pb(1^i^,2^i^) 155.04(2), 155.123(19), Pb(1,2)⋅⋅⋅Au–Sb(2,3) 58.73(2), 59.175(15), Pb(1,2)⋅⋅⋅Au–Sb(3,2) 110.91(2), 111.25(2). Symmetry code: i=1−*x*, 1−*y*, 1−*z*. Figures of compounds **2** and **3** are provided in the Supporting Information.

Compounds **2**–**4** were also characterized by means of SC‐XRD^18^ and μ‐XFS. Additionally, ESI mass spectra were recorded on fresh solutions of single‐crystals, which indicated different degrees of fragmentation under ESI‐MS conditions for the three different species, notably also producing (SnBi_3_)^−^ upon redissolving the single‐crystals in the case of **2**. Yet, the sensitivity of the compounds did not allow for their transfer into the gas phase as a whole. The anions in **2**–**4** are isostructural to each other and with the anion of the above‐mentioned copper homologue of compound **3**. They thus represent dimers of trimetallic 9‐vertex cages, which can be understood as heterometallic superatoms, according to comprehensive quantum chemical studies carried out on the anion {[CuSn_5_Sb_3_]_2_}^4−^.7d The molecular structure of {[AuPb_5_Sb_3_]_2_}^4−^ in compound **4** is shown in Figure 5 as an example.

In this case, the assignment of Pb and Sb atoms was unambiguous and not affected by positional disorder. Pb and Sb atoms occupy the same positions as the Sn and Sb atoms, respectively, in the previously reported Cu cluster, which serves to confirm the previous assignment that was made based on perturbation theory for the quoted cluster. The Au atoms are coordinated by two Pb and two Sb atoms each, in a distorted square planar fashion, with the Au–Pb bonds being by 0.12–0.17 Å longer than the Au–Sb bonds. Two additional, yet significantly longer (weaker) Au⋅⋅⋅Pb interactions complement the arrangement into a distorted trigonal prismatic environment.

Although it is not possible to calculate reaction energies of the reorganization to form the 8‐vertex units that coordinate the Au atoms in compounds **2**–**4**, starting out from the precursor units, as these reactions take place under overall oxidation of the main group elements, we suspect that the lower preference for the reorganizations shown in Equation (1) allows for such alternative fragmentation and rearrangement reactions to take place instead. One should additionally take into consideration that the bonding within the 8‐vertex units has an even more delocalized character than in the *pseudo*‐tetrahedral anions,^27^ which may explain their preference to form with increasing atomic number (and thus the metal character) of the involved atom types.

According to DFT calculations of the anions in **2**–**4**, and also of the yet “missing” Au/Pb/Bi analogue, we can make the following conclusions: First, the atom assignment of tetrel and pentel atoms clearly is preferred in the way shown in Figure 5. Second, according to population analyses (Table S10) and the inspection of localized molecular orbitals (Figures S33–S35), the Au atoms do not undergo notable Au⋅⋅⋅Au interactions here—similar to the observations made for compound **1**—but prefer the formation of heterometallic (mainly three‐center) bonds. As the bonding situation in **2**–**4** is qualitatively the same as in the copper analogue of compound **3**, we do not detail it here, but refer to the respective literature.7d

Notably, all our attempts failed so far to grow crystals from reactions with the heaviest homologue in this series, (Pb_2_Bi_2_)^2−^, or to find any further evidence for the formation of a cluster anion comprising Au besides the said main group metals. As both elemental combinations, Au/Bi and Au/Pb, have been realized (see below), and as other clusters with the elemental combinations Ln/Pb/Bi,7e Pd/Pb/Bi,7f Ni/Pb/Bi,7g Zn/Pb/Bi,7g and U/Pb/Bi^10^ have been known, and as the tendency of the (Pb_2_Bi_2_)^2−^ anion to reorganize seems to be similar to that of (Sn_2_Bi_2_)^2−^ (see Figure 4), this observation is unexpected. It is therefore subject to ongoing work. Finally, it is worth noting here that Au atoms were successfully introduced into several other heterometallic and intermetalloid complexes or clusters beside those mentioned above. In these molecules, which are either anionic, cationic or uncharged, the Au atoms exhibit a diversity of coordination environments.^28^, ^29^, ^30^, ^31^

## Conclusion

In summary, we presented two new types of intermetalloid cluster architectures comprising gold atoms, resembling a yet unprecedented 22‐atomic supertetrahedral arrangement and a series of dimeric clusters of ternary 9‐vertex cages. Besides experimental work including X‐ray diffraction, EDS/μ‐XFS, and ESI mass spectrometry, quantum chemical studies were applied to confirm the composition and to determine atomic positions in the structures. In addition, the calculations verified that intense Au–Tt or Au–Pn bonding outplays Au⋅⋅⋅Au interactions in these cases, and they served to illustrate that the lightest congeners used in ternary intermetalloid cluster syntheses, (Ge_2_P_2_)^2−^ and (Ge_2_As_2_)^2−^ readily undergo reorganization reactions in solution to provide (Tt_3_Pn)^3−^ units for the cluster formation, while other elemental combinations prefer other rearrangement pathways.

These new insights allowed us to push the limits of Zintl chemistry towards new elemental combinations and architectures, which will be the base for future studies about such uncommon metal nanoclusters and their formation processes.

## Conflict of interest

The authors declare no conflict of interest.

## Supporting information

As a service to our authors and readers, this journal provides supporting information supplied by the authors. Such materials are peer reviewed and may be re‐organized for online delivery, but are not copy‐edited or typeset. Technical support issues arising from supporting information (other than missing files) should be addressed to the authors.

Supplementary

## References

[1a] M. Scheer , G. Balázs , A. Seitz , Chem. Rev. 2010, 110, 4236–4256; 20438122 10.1021/cr100010e

[1b] M. Melaimi , R. Jazzar , M. Soleilhavoup , G. Bertrand , Angew. Chem. Int. Ed. 2017, 56, 10046–10068; 10.1002/anie.201702148 28376253

[1c] T. Li , S. Kaercher , P. W. Roesky , Chem. Soc. Rev. 2014, 43, 42–57. 23945727 10.1039/c3cs60163c

[2] F. Spitzer , M. Sierka , M. Latronico , P. Mastrorilli , A. V. Virovets , M. Scheer , Angew. Chem. Int. Ed. 2015, 54, 4392–4396; 10.1002/anie.201411451 25677593

[3] C. Schwarzmaier , A. Schindler , C. Heindl , S. Scheuermayer , E. V. Peresypkina , A. V. Virovets , M. Neumeier , R. Gschwind , M. Scheer , Angew. Chem. Int. Ed. 2013, 52, 10896–10899; 10.1002/anie.201306146 24000137

[4a] M. Seidl , G. Balázs , M. Scheer , Chem. Rev. 2019, 119, 8406–8434; 30900440 10.1021/acs.chemrev.8b00713

[4b] A. E. Seitz , F. Hippauf , W. Kremer , S. Kaskel , M. Scheer , Nat. Commun. 2018, 9, 361; 29367623 10.1038/s41467-017-02735-2 PMC5783940

[4c] M. Schmidt , A. E. Seitz , M. Eckhardt , G. Balázs , E. V. Peresypkina , A. V. Virovets , F. Riedlberger , M. Bodensteiner , E. M. Zolnhofer , K. Meyer , M. Scheer , J. Am. Chem. Soc. 2017, 139, 13981–13984; 28933848 10.1021/jacs.7b07354

[4d] C. Schoo , S. Bestgen , A. Egeberg , J. Seibert , S. N. Konchenko , C. Feldmann , P. W. Roesky , Angew. Chem. Int. Ed. 2019, 58, 4386–4389; 10.1002/anie.201813370 30614173

[5] B. M. Cossairt , M.-C. Diawara , C. C. Cummins , Science 2009, 323, 602. 19179522 10.1126/science.1168260

[6] R. J. Wilson , N. Lichtenberger , B. Weinert , S. Dehnen , Chem. Rev. 2019, 119, 8506–8554. 31136158 10.1021/acs.chemrev.8b00658

[7a] N. Lichtenberger , W. Massa , S. Dehnen , Angew. Chem. Int. Ed. 2019, 58, 3222–3226; 10.1002/anie.201812473 30614170

[7b] R. J. Wilson , F. Hastreiter , K. Reiter , P. Büschelberger , R. Wolf , R. Gschwind , F. Weigend , S. Dehnen , Angew. Chem. Int. Ed. 2018, 57, 15359–15363; 10.1002/anie.201807180 30270504

[7c] S. Mitzinger , J. Bandemehr , K. Reiter , S. J. McIndoe , X. Xie , F. Weigend , J. F. Corrigan , S. Dehnen , Chem. Commun. 2018, 54, 1421–1424; 10.1039/c7cc08348c 29303170

[7d] R. J. Wilson , L. Broeckaert , F. Spitzer , F. Weigend , S. Dehnen , Angew. Chem. Int. Ed. 2016, 55, 11775–11780; 10.1002/anie.201603455 27558912

[7e] R. Ababei , W. Massa , B. Weinert , P. Pollak , X. Xie , R. Clérac , F. Weigend , S. Dehnen , Chem. Eur. J. 2015, 21, 386–394; 25412590 10.1002/chem.201404904

[7f] R. Ababei , W. Massa , K. Harms , X. Xie , F. Weigend , S. Dehnen , Angew. Chem. Int. Ed. 2013, 52, 13544–13548; 10.1002/anie.201307795 24346937

[7g] R. Ababei , J. Heine , M. Hołyńska , G. Thiele , B. Weinert , X. Xie , F. Weigend , S. Dehnen , Chem. Commun. 2012, 48, 11295–11297; 10.1039/c2cc35318k 22962663

[7h] F. Lips , S. Dehnen , Angew. Chem. Int. Ed. 2011, 50, 955–959; 10.1002/anie.201005496 21246699

[7i] F. Lips , R. Clérac , S. Dehnen , J. Am. Chem. Soc. 2011, 133, 14168–14171; 21834524 10.1021/ja203302t

[7j] F. Lips , S. Dehnen , Angew. Chem. Int. Ed. 2009, 48, 6435–6438; 10.1002/anie.200902249 19637177

[8] N. Lichtenberger , N. Spang , A. Eichhöfer , S. Dehnen , Angew. Chem. Int. Ed. 2017, 56, 13253–13258; 10.1002/anie.201707632 28834005

[9a] B. Weinert , F. Müller , K. Harms , S. Dehnen , Angew. Chem. Int. Ed. 2014, 53, 11979–11983; 10.1002/anie.201407288 25212185

[9b] B. Weinert , F. Weigend , S. Dehnen , Chem. Eur. J. 2012, 18, 13589–13595; 22996133 10.1002/chem.201202369

[9c] F. Lips , M. Hołyńska , R. Clérac , U. Linne , I. Schellenberg , R. Pöttgen , F. Weigend , S. Dehnen , J. Am. Chem. Soc. 2012, 134, 1181–1191; 22111958 10.1021/ja209226b

[9d] F. Lips , R. Clérac , S. Dehnen , Angew. Chem. Int. Ed. 2011, 50, 960–964; 10.1002/anie.201005655 21246700

[10] N. Lichtenberger , R. J. Wilson , A. R. Eulenstein , W. Massa , R. Clerac , F. Weigend , S. Dehnen , J. Am. Chem. Soc. 2016, 138, 9033–9036. 27392253 10.1021/jacs.6b04363

[11a] S. Mitzinger , L. Broeckaert , W. Massa , F. Weigend , S. Dehnen , Nat. Commun. 2016, 7, 10480–10490; 26805602 10.1038/ncomms10480 PMC4737759

[11b] B. Weinert , S. Mitzinger , S. Dehnen , Chem. Eur. J. 2018, 24, 8470–8490; 29466607 10.1002/chem.201704904

[11c] crypt-222=4,7,13,16,21,24-Hexaoxa-1,10-diazabicyclo[8.8.8]-hexacosane.

[12a] M. Ichinohe , M. Toyoshima , R. Kinjo , A. Sekiguchi , J. Am. Chem. Soc. 2003, 125, 13328–13329; 14583007 10.1021/ja0305050

[12b] Y. Heider , P. Willmes , V. Huch , M. Zimmer , D. Scheschkewitz , J. Am. Chem. Soc. 2019, 141, 19498–19504. 31726826 10.1021/jacs.9b11181

[13a] M. Waibel , F. Kraus , S. Scharfe , B. Wahl , T. F. Fässler , Angew. Chem. Int. Ed. 2010, 49, 6611–6615; 10.1002/anie.201002153 20677294

[13b] M. Waibel , G. Raudaschl-Sieber , T. F. Fässler , Chem. Eur. J. 2011, 17, 13391–13394; 22034175 10.1002/chem.201102095

[13c] S. Stegmaier , M. Waibel , A. Henze , L. A. Jantke , A. J. Karttunen , T. F. Fässler , J. Am. Chem. Soc. 2012, 134, 14450–14460; 22867109 10.1021/ja304251t

[13d] T. Henneberger , W. Klein , J. V. Dums , T. F. Fässler , Chem. Commun. 2018, 54, 12381–12384; 10.1039/c8cc06843g 30328416

[13e] C. B. Benda , M. Waibel , T. Kochner , T. F. Fässler , Chem. Eur. J. 2014, 20, 16738–16746. 25318859 10.1002/chem.201404594

[14a] F. Fendt , C. Koch , S. Gartner , N. Korber , Dalton Trans. 2013, 42, 15548–15550; 24067942 10.1039/c3dt51932e

[14b] V. Queneau , S. C. Sevov , J. Am. Chem. Soc. 1997, 119, 8109–8110.

[15] U. Zachwieja , J. Müller , J. Wlodarski , Z. Anorg. Allg. Chem. 1998, 624, 853–858.

[16] D. P. Huang , J. D. Corbett , Inorg. Chem. 1998, 37, 5007–5010. 11670669 10.1021/ic980579q

[17] F.-X. Pan , L. J. Li , Z. M. Sun , Chin. J. Struct. Chem. 2016, 35, 1099–1106.

[18] Deposition Number(s) 2008058 (for **1**), 2008055 (for **2**), 2008057 (for **3**), and 2008056 (for **4**) contain the supplementary crystallographic data for this paper. These data are provided free of charge by the joint Cambridge Crystallographic Data Centre and Fachinformationszentrum Karlsruhe Access Structures service www.ccdc.cam.ac.uk/structures.

[19] TURBOMOLE V7.4.1 2019, a development of University of Karlsruhe and Forschungszentrum Karlsruhe GmbH, 1989–2007, TURBOMOLE GmbH, since 2007; available from http://www.turbomole.com.

[20] S. G. Balasubramani , G. P. Chen , S. Coriani , M. Diedenhofen , M. S. Frank , Y. J. Franzke , F. Furche , R. Grotjahn , M. E. Harding , C. Hättig , A. Hellweg , B. Helmich-Paris , C. Holzer , U. Huniar , M. Kaupp , A. M. Khah , S. K. Khani , T. Müller , F. Mack , B. D. Nguyen , S. M. Parker , E. Perlt , D. Rappoport , K. Reiter , S. Roy , M. Rückert , G. Schmitz , M. Sierka , E. Tapavicza , D. P. Tew , C. van Wüllen , V. K. Voora , F. Weigend , A. Wodyński , and J. M. Yu , J. Chem. Phys. 2020, 152, 184107. 32414256 10.1063/5.0004635 PMC7228783

[21] J. Tao , J. P. Perdew , V. N. Staroverov , G. E. Scuseria , Phys. Rev. Lett. 2003, 91, 146401. 14611541 10.1103/PhysRevLett.91.146401

[22a] F. Weigend , R. Ahlrichs , Phys. Chem. Chem. Phys. 2005, 7, 3297–3305; 16240044 10.1039/b508541a

[22b] F. Weigend , A. Baldes , J. Chem. Phys. 2010, 133, 174102. 21054001 10.1063/1.3495681

[23] F. Weigend , Phys. Chem. Chem. Phys. 2006, 8, 1057–1065. 16633586 10.1039/b515623h

[24a] B. Metz , H. Stoll , M. Dolg , J. Chem. Phys. 2000, 113, 2563–2569;

[24b] D. Figgen , G. Rauhaut , M. Dolg , H. Stoll , Chem. Phys. 2005, 311, 227–244.

[25a] F. Weigend , C. Schrodt , R. Ahlrichs , J. Chem. Phys. 2004, 121, 10380–10384; 15549917 10.1063/1.1811079

[25b] F. Weigend , C. Schrodt , Chem. Eur. J. 2005, 11, 3559–3564; 15809984 10.1002/chem.200500028

[25c] F. Weigend , J. Chem. Phys. 2014, 141, 134103. 25296780 10.1063/1.4896658

[26] L. Guggolz , S. Dehnen , Chem. Eur. J. 2020, 10.1002/chem.202001379. PMC7540718 32285972

[27] R. J. Wilson , F. Weigend , S. Dehnen , Angew. Chem. Int. Ed. 2020, 10.1002/anie.202002863; PMC7496391 32449980

[28a] U. Müller , A. Isaeva , J. Richter , M. Knies , M. Ruck , Eur. J. Inorg. Chem. 2016, 3580–3584;

[28b] F. S. Geitner , W. Klein , T. F. Fässler , Dalton Trans. 2017, 46, 5796–5800; 28426036 10.1039/c7dt00754j

[28c] F. S. Geitner , T. F. Fässler , Eur. J. Inorg. Chem. 2016, 2688–2691;

[28d] M. Binder , C. Schrenk , T. Block , R. Pöttgen , A. Schnepf , Chem. Commun. 2017, 53, 11314–11317; 10.1039/c7cc07029b 28967017

[28e] C. Liu , L. J. Li , X. Jin , J. E. McGrady , Z. M. Sun , Inorg. Chem. 2018, 57, 3025–3034. 29512998 10.1021/acs.inorgchem.7b02620

[29a] L. J. Schiegerl , F. S. Geitner , C. Fischer , W. Klein , T. F. Fässler , Z. Anorg. Allg. Chem. 2016, 642, 1419–1426;

[29b] C. Schenk , A. Schnepf , Angew. Chem. Int. Ed. 2007, 46, 5314–5316; 10.1002/anie.200700530 17579905

[29c] C. Schenk , F. Henke , G. Santiso-Quinones , I. Krossing , Dalton Trans. 2008, 4436–4441; 18698446 10.1039/b717506j

[29d] F. S. Geitner , M. A. Giebel , A. Pothig , T. F. Fässler , Molecules 2017, 22, 1204; 10.3390/molecules22071204 PMC6152075 28753928

[29e] C. M. Knapp , C. S. Jackson , J. S. Large , A. L. Thompson , J. M. Goicoechea , Inorg. Chem. 2011, 50, 4021–4028; 21425807 10.1021/ic102516g

[29f] N. K. Chaki , S. Mandal , A. C. Reber , M. C. Qian , H. M. Saavedra , P. S. Weiss , S. N. Khanna , A. Sen , ACS Nano 2010, 4, 5813–5818; 20882982 10.1021/nn101640r

[29g] M. C. Qian , A. C. Reber , A. Ugrinov , N. K. Chaki , S. Mandal , H. M. Saavedra , S. N. Khanna , A. Sen , P. S. Weiss , ACS Nano 2010, 4, 235–240; 20038127 10.1021/nn9010297

[29h] A. Spiekermann , S. D. Hoffmann , F. Kraus , T. F. Fässler , Angew. Chem. Int. Ed. 2007, 46, 1638–1640; 10.1002/anie.200602003 17230593

[30a] B. Wahl , M. Erbe , A. Gerisch , L. Kloo , M. Ruck , Z. Anorg. Allg. Chem. 2009, 635, 743–752;

[30b] B. Wahl , L. Kloo , M. Ruck , Angew. Chem. Int. Ed. 2008, 47, 3932–3935; 10.1002/anie.200800142 18412201

[30c] B. Wahl , M. Ruck , Z. Anorg. Allg. Chem. 2008, 634, 2267–2275.

[31a] F.-X. Pan , L.-J. Li , Y.-J. Wang , J.-C. Guo , H.-J. Zhai , L. Xu , Z.-M. Sun , J. Am. Chem. Soc. 2015, 137, 10954–10957; 26275027 10.1021/jacs.5b07730

[31b] I. A. Popov , F.-X. Pan , X. R. You , L. J. Li , E. Matito , C. Liu , H. J. Zhai , Z. M. Sun , A. I. Boldyrev , Angew. Chem. Int. Ed. 2016, 55, 15344–15346; 10.1002/anie.201609497 27862764

[31c] A. Spiekermann , S. D. Hoffmann , T. F. Fässler , I. Krossing , U. Preiss , Angew. Chem. Int. Ed. 2007, 46, 5310–5313; 10.1002/anie.200603389 17546713

[31d] L.-J. Li , F.-X. Pan , F.-Y. Li , Z.-F. Chen , Z.-M. Sun , Inorg. Chem. Front. 2017, 4, 1393–1396.

