# Supplementary material for: Atom Exchange Versus Reconstruction: (GexAs4−x)x− (x=2, 3) as Building Blocks for the Supertetrahedral Zintl Cluster [Au6(Ge3As)(Ge2As2)3]3−
Source: Angew Chem Int Ed Engl. 2020 Aug 4;59(38):16638–43. doi: 10.1002/anie.202008108 (PMC7540319; doi:10.1002/anie.202008108)
Supplement: Supplementary file 1 — Supplementary [file ANIE-59-16638-s001.pdf]

Angewandte Chemie
Eine Zeitschrift der Gesellschaft Deutscher Chemiker

Supporting Information

Atom Exchange Versus Reconstruction: $(Ge_xAs_{4-x})^{x-}$ ($x = 2, 3$) as Building Blocks for the Supertetrahedral Zintl Cluster $[Au_6(Ge_3As)(Ge_2As_2)_3]^{3-}$

*Fuxing Pan, Lukas Guggolz, Florian Weigend, and Stefanie Dehnen**

anie_202008108_sm_miscellaneous_information.pdf

1. Synthesis Details

1.1 General

All manipulations and reactions were performed under dry Ar atmosphere using standard Schlenk or glovebox techniques. All solvents were dried and freshly distilled prior to use, crypt-222 (Merck)[1] was dried in vacuo for at least 18 hours. [K(crypt-222)]$_2$(Tt$_2$Pn$_2$)·en (Tt/Pn = Ge/As, Sn/Bi, Sn/Sb and Pb/Sb) were prepared according to literatures, respectively.[2] [(PPh$_3$)AuMe] was commercially available from Aldrich. Samples were shielded from ambient light throughout cluster syntheses.

1.2 Syntheses

1.2.1. Synthesis of [K(crypt-222)]$_3$[Au$_6$(Ge$_3$As)(Ge$_2$As$_2$)$_3$]·en·2tol (1)

80 mg (0.067 mmol) of [K(crypt-222)]$_2$(Ge$_2$As$_2$)·en and 48 mg (0.10 mmol) of [(PPh$_3$)AuMe] were combined in a Schlenk tube and dissolved in en (3 mL). The initially red solution slowly changed to dark brown. The reaction mixture was allowed to stir for 3 hours. The resulting dark brown solution was filtered through a standard glass frit, carefully layered with toluene (3 mL), and stored for crystallization at 5 °C. After 15 days, block-like crystals of compound **1** formed at the wall of the Schlenk tube in appr. 6 % yield (Figure S1).

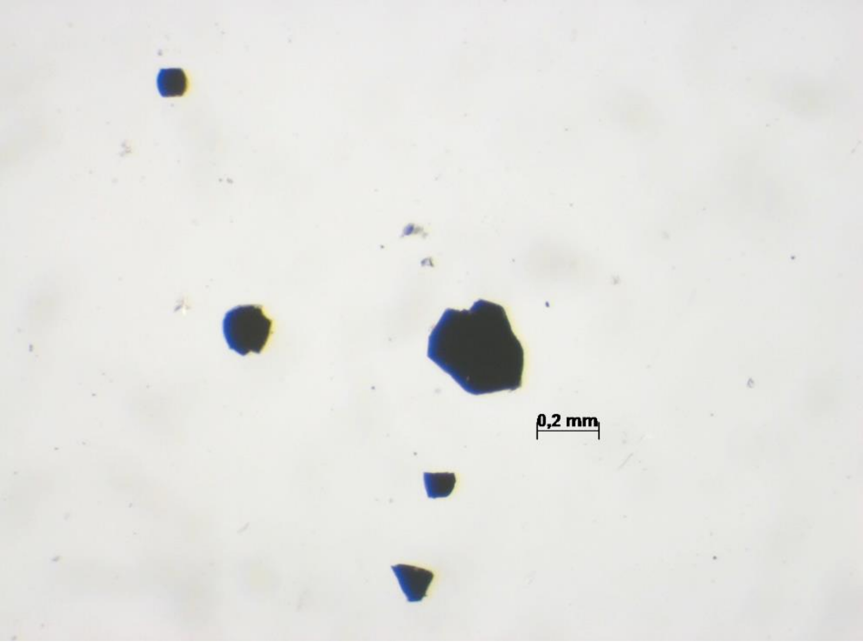

Figure S1. Crystal photograph of **1**.

1.2.2. Synthesis of [K(crypt-222)]$_4${[AuSn$_5$Bi$_3$]$_2$}·2py (2)

80 mg (0.052 mmol) of [K(crypt-222)]$_2$(Sn$_2$Bi$_2$)·en and 25 mg (0.052 mmol) of [(PPh$_3$)AuMe] were combined in a Schlenk tube and dissolved in pyridine (3 mL). The reaction mixture was allowed to stir for 3 hours. The resulting dark brown solution was filtered through a standard glass frit, carefully layered with toluene (3 mL), and stored for crystallization at 5 °C. After 15 days, plate-like crystals of compound **2** formed at the wall of the Schlenk tube in appr. 15 % yield (Figure S2).

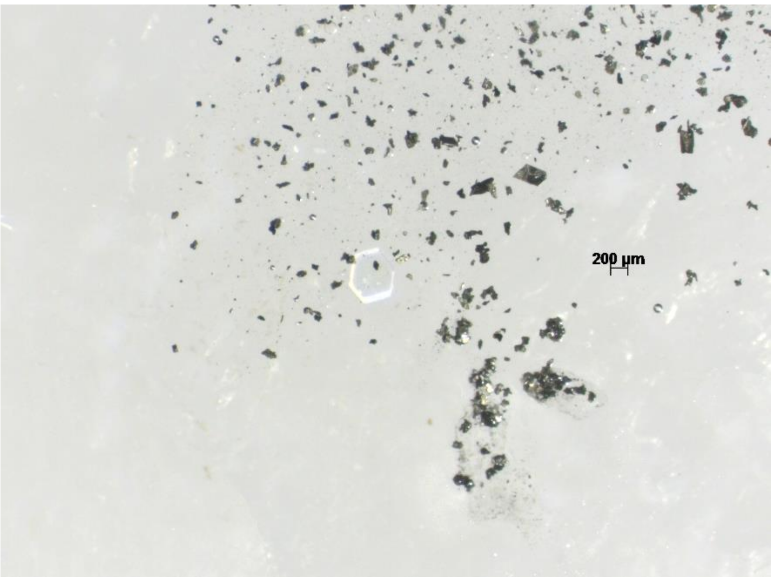

Figure S2. Crystal photograph of **2**.

1.2.3. Synthesis of $[K(crypt\text{-}222)]_4\{[AuSn_5Sb_3]_2\}\cdot 2py$ (3)

80 mg (0.058 mmol) of $[K(crypt\text{-}222)]_2(Sn_2Sb_2)\cdot en$ and 28 mg (0.058 mmol) of $[(PPh_3)AuMe]$ were combined in a Schlenk tube and dissolved in pyridine (3 mL). The reaction mixture was allowed to stir for 3 hours. The resulting dark brown solution was filtered through a standard glass frit, carefully layered with toluene (3 mL), and stored for crystallization at 5 °C. After 15 days, plate-like crystals of compound **3** formed at the wall of the Schlenk tube in appr. 25 % yield (Figure S3).

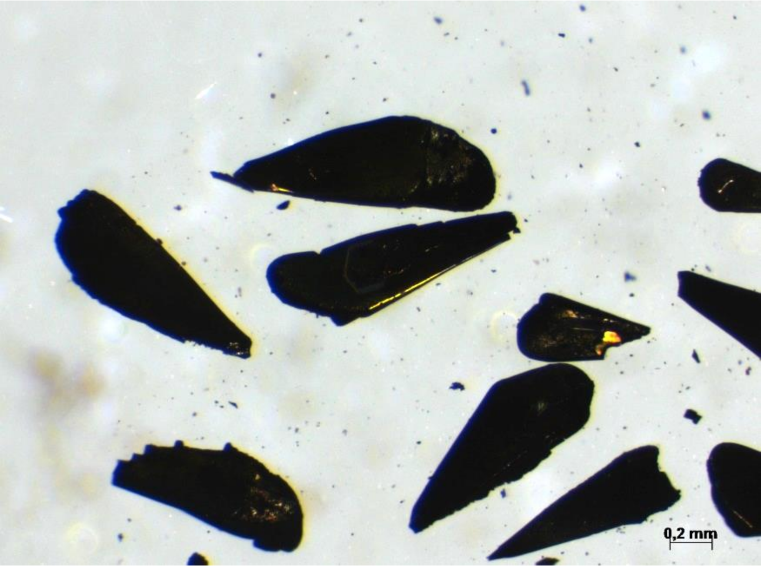

Figure S3. Crystal photograph of **3**.

1.2.4. Synthesis of $[K(crypt\text{-}222)]_4\{[AuPb_5Sb_3]_2\}\cdot 3en$ (4)

80 mg (0.052 mmol) of $[K(crypt\text{-}222)]_2(Sn_2Sb_2)\cdot en$ and 24 mg (0.052 mmol) of $[(PPh_3)AuMe]$ were combined in a Schlenk tube and dissolved in en (3 mL). The reaction mixture was allowed to stir for 3 hours. The resulting dark brown solution was filtered through a standard glass frit, carefully layered with toluene (3 mL), and stored for crystallization at 5 °C. After 15 days, needle-like crystals of compound **4** formed at the wall of the Schlenk tube in appr. 31 % yield (Figure S4).

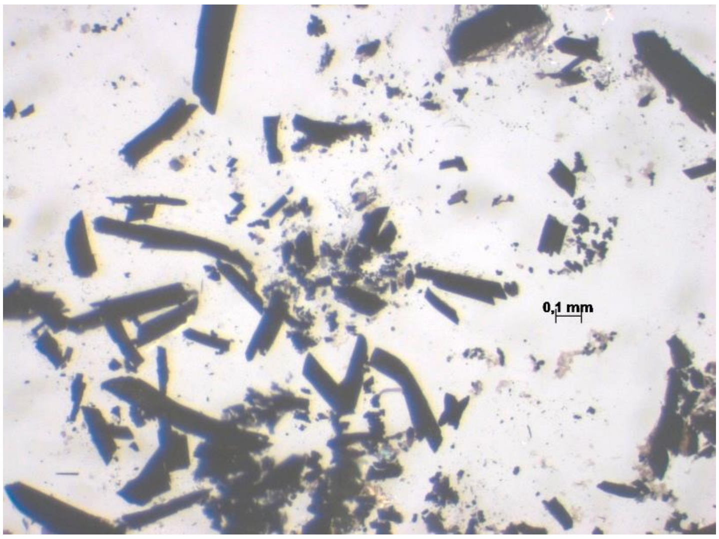

Figure S4. Crystal photograph of **4**.

2. Single crystal X-ray crystallography (SCXRD)

2.1. Single Crystal X-ray Diffraction

The data for the X-ray structural analyses were collected at T =100.0 K with Mo-Kα-radiation (λ = 0.71073 Å) on a Bruker D8Quest with a CMOS detector for **1** and **3**, and on an area detector systems Stoe IPDS/2T for **4**, and at T = 100(2) K with Cu Kα-radiation (λ = 1.5418 Å) on a STOE STADIVARI with an image plate detector for **2**. The structure was solved by methods of SHELXT from SHELXL-2018/136,[3] and refined by full matrix least-squares methods against F^2 with the SHELXL program.[4] All hydrogen atoms were kept riding on calculated positions with isotropic displacement parameters U = 1.2 Ueq of the bonding partners. In the refinement of compounds **1** and **5**, atoms Ge/As or Sn/Sb were assigned according to the result of the perturbation theory results based on the DFT investigations. The relatively large tendency for disorder of the crypt-222 molecules in compounds **2** and **3** required the application of restraints to this large number of atoms (the alternative application of the back-Fourier-transform method did not produce more reliable results). Crystallographic data (excluding structure factors) for the structure reported in this paper have been deposited with the Cambridge Crystallographic Data Centre as supplementary publication no. CCDC-2008055 – CCDC-2008058. Copies of the data can be obtained free of charge on application to CCDC, 12 Union Road, Cambridge CB2 1EZ, UK [fax.: (internat.) + 44 1223/336-033; e-mail: deposit@ccdc.cam.ac.uk]. The crystal data and experimental parameters of the structure determinations are collected in Table S1, structural parameters are given in Tables S2-S5. Structure Figures (Figures S5-S8) were created with Diamond 4.[5]

2.2. Details of Structures

Table S1. Crystal data and details of the structure determinations of 4 compounds.

Compound	1	2	3	4
Empirical formula	$C_{70}H_{132}As_7Au_6Ge_9K_3N_8O_{18}$	$C_{92}H_{140}Au_2Bi_6K_4N_{12}O_{24}Sn_{10}$	$C_{94}H_{148}Au_2K_4N_{10}O_{24}Sb_6Sn_{10}$	$C_{78}H_{172}Au_2K_4N_{14}O_{24}Pb_{10}Sb_6$
Formula weight /g mol^{-1}	3880.72	4789.26	4269.95	5043.02
Temperature /K	100	100	100	100
Crystal system	monoclinic	monoclinic	monoclinic	monoclinic
Space group	*C*2/c	*P*2$_1$/n	*P*2$_1$/n	P2$_1$/c
a /Å	42.0518(19)	19.480(4)	19.4134(11)	15.610(3)
b /Å	23.7224(11)	15.790(3)	15.6901(9)	26.030(5)
c /Å	26.8265(12)	21.890(4)	21.9347(12)	17.170(3)
α /°	90	90	90	90
β /°	120.5730(10)	98.35(3)	98.094(2)	114.05(3)
γ /°	90	90	90	90
Volume /Å^3	23041.0(18)	6662(2)	6614.7(6)	6371(3)
Z	8	2	2	2
ρ_{calc} g/cm^3	2.237	2.388	2.144	2.629
μ /mm^{-1}	12.368	12.107	5.448	16.888
F(000)	14520	4400	4028	4580
Radiation	MoKα (λ = 0.71073)	MoKα (λ = 0.71073)	MoKα (λ = 0.71073)	MoKα (λ = 0.71073)
2Θ range for data collection/°	4.606 to 56.768	3.334 to 53.246	4.562 to 49.656	5.05 to 53.552
Index ranges	$-56 \le h \le 56$, $-31 \le k \le 31$, $-35 \le l \le 35$	$-24 \le h \le 10$, $-19 \le k \le 19$, $-26 \le l \le 26$	$-22 \le h \le 22$, $-18 \le k \le 18$, $-25 \le l \le 25$	$-19 \le h \le 19$, $-32 \le k \le 32$, $-21 \le l \le 21$
Reflections collected	353257	41523	195355	47750
Independent reflections	28837 [R_{int} = 0.0609, R_{sigma} = 0.0308]	13272 [R_{int} = 0.0883, R_{sigma} = 0.1592]	11375 [R_{int} = 0.0781, R_{sigma} = 0.0285]	13488 [R_{int} = 0.0997, R_{sigma} = 0.0836]
Data/restraints/parameters	28837/0/1094	13272/1270/676	11375/546/694	13488/0/625
Goodness-of-fit on F^2	1.012	0.73	1.12	0.95
Final R indexes [$I \ge 2\sigma$ (I)]	R_1 = 0.0298, wR_2 = 0.0583	R_1 = 0.0509, wR_2 = 0.1122	R_1 = 0.0474, wR_2 = 0.1147	R_1 = 0.0351, wR_2 = 0.0735
Final R indexes [all data]	R_1 = 0.0511, wR_2 = 0.0650	R_1 = 0.1357, wR_2 = 0.1374	R_1 = 0.0667, wR_2 = 0.1287	R_1 = 0.0603, wR_2 = 0.0780
Largest diff. peak/hole /e Å^{-3}	1.39/−1.13	1.41/−1.74	2.03/−1.42	2.37/−1.90
CCDC deposition number	2008058	2008055	2008057	2008056

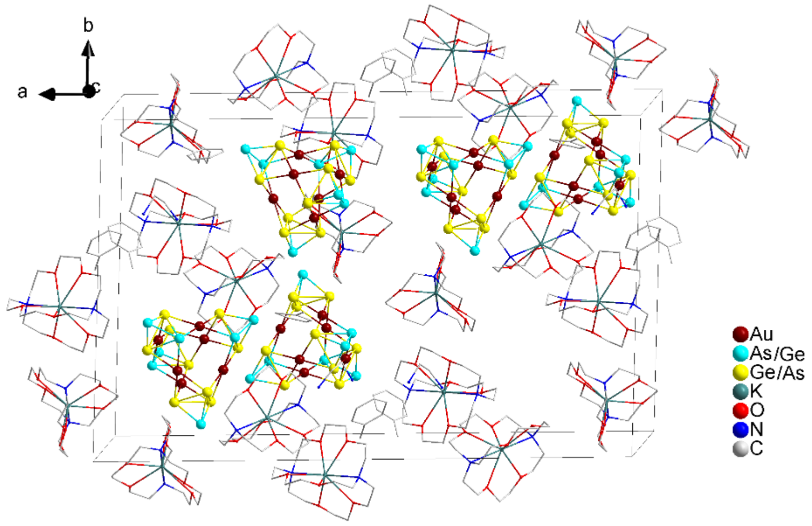

Figure S5. Unit cell of compound **1**.

Table S2. Selected interatomic distances (in Å) of the experimental and optimized structures of the anion in 1.

Bond type	Measured bond length	Calculated bond length
Au1-As/Ge3	2.5497(6)	2.594
Au1-Ge/As1	2.5662(6)	2.573
Au1-Ge/As2	2.5540(6)	2.558
Au1-Ge/As5	2.5629(6)	2.607
Au2-As/Ge5	2.5546(5)	2.599
Au2-Ge/As2	2.5548(5)	2.562
Au2-Ge/As3	2.5629(5)	2.577
Au2-Ge/As7	2.5644(5)	2.612
Au3-As/Ge7	2.5589(5)	2.595
Au3-Ge/As1	2.5493(5)	2.574
Au3-Ge/As3	2.5535(5)	2.560
Au3-Ge/As9	2.5438(5)	2.611
Au4-As/Ge3	2.5582(6)	2.582
Au4-As/Ge7	2.5474(5)	2.578
Au4-Ge/As4	2.5465(6)	2.570
Au4-Ge/As8	2.5615(6)	2.573
Au5-Ge/As4	2.5513(5)	2.580
Au5-Ge/As5	2.5533(6)	2.585
Au5-Ge/As6	2.5584(5)	2.582
Au5-Ge/As7	2.5369(5)	2.583
Au6-As/Ge5	2.5609(6)	2.594
Au6-Ge/As6	2.5474(5)	2.604
Au6-Ge/As8	2.5599(6)	2.554
Au6-Ge/As9	2.5616(5)	2.574
As/Ge1-Ge/As1	2.4581(8)	2.473
As/Ge1-Ge/As2	2.4467(8)	2.483
As/Ge1-Ge/As3	2.4500(7)	2.480
As/Ge2-As/Ge3	2.4572(8)	2.440
As/Ge2-Ge/As4	2.4361(8)	2.484
As/Ge2-Ge/As5	2.4523(8)	2.509
As/Ge3-Ge/As4	2.8053(7)	2.797
As/Ge3-Ge/As5	2.8036(8)	2.757
As/Ge4-As/Ge5	2.4470(7)	2.448
As/Ge4-Ge/As6	2.4561(7)	2.485
As/Ge4-Ge/As7	2.4453(7)	2.503
As/Ge5-Ge/As6	2.8020(7)	2.768
As/Ge5-Ge/As7	2.8071(7)	2.755
As/Ge6-As/Ge7	2.4559(7)	2.443
As/Ge6-Ge/As8	2.4514(7)	2.483
As/Ge6-Ge/As9	2.4396(7)	2.503
As/Ge7-Ge/As8	2.8170(7)	2.801
As/Ge7-Ge/As9	2.8052(7)	2.751
Ge/As1-Ge/As2	2.7954(7)	2.875
Ge/As1-Ge/As3	2.8099(7)	2.872
Ge/As2-Ge/As3	2.8016(7)	2.872
Ge/As4-Ge/As5	2.7879(8)	2.843
Ge/As6-Ge/As7	2.8279(7)	2.848
Ge/As8-Ge/As9	2.7883(7)	2.880

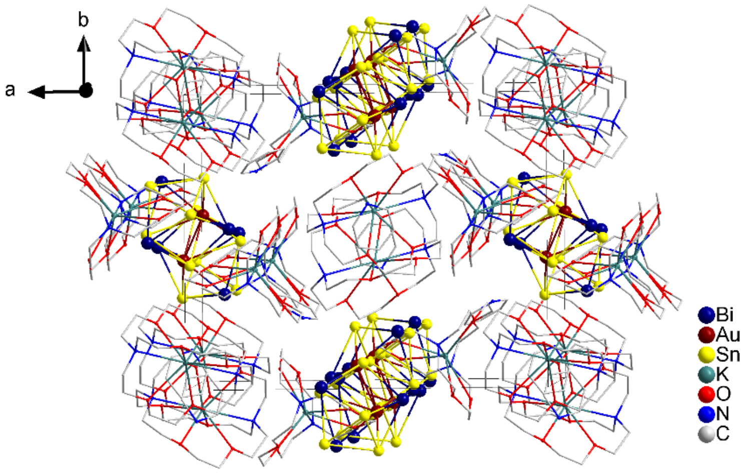

Figure S6. Unit cell of compound **2**.

Table S3. Selected interatomic distances (in Å) of the experimental and optimized structures of the anion in 2.

Bond type	Measured bond length	Calculated bond length
Sn1–Sn2	3.1558(18)	3.157
Sn3–Sn4	2.9553(19)	2.986
Sn3–Sn5	3.0627(19)	3.128
Sn4–Sn5	3.0606(17)	3.129
Bi1–Sn1	3.0481(15)	3.067
Bi1–Sn2	3.0485(16)	3.069
Bi1–Sn3	3.0080(18)	3.048
Bi1–Sn4	3.0405(15)	3.044
Bi2–Sn1	2.9913(16)	3.004
Bi2–Sn3	2.9909(16)	3.025
Bi2–Sn5	3.0350(16)	3.045
Bi3–Sn2	2.9673(15)	3.004
Bi3–Sn4	2.9827(16)	3.023
Bi3–Sn5	3.0115(18)	3.043
Au1···Sn1	3.1665(15)	3.135
Au1–Sn1^{i}	2.7609(16)	2.816
Au1···Sn2	3.2123(14)	3.132
Au1–Sn2^{i}	2.7766(14)	2.818
Au1–Bi2	2.8448(14)	2.846
Au1–Bi3	2.8507(11)	2.845
Au1···Au1^{i}	2.8833(16)	2.898
Au1···Sn5	2.9584(15)	2.904

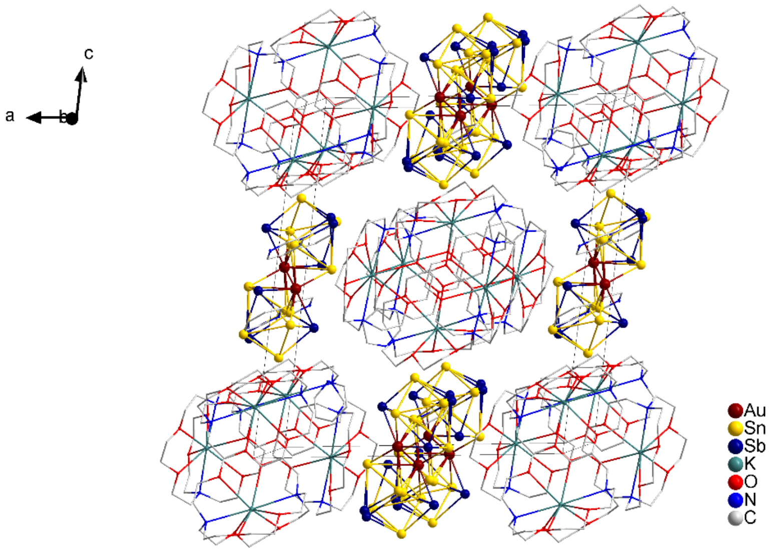

Figure S7. Unit cell of compound **3**.

Table S4. Selected interatomic distances (in Å) of the experimental and optimized structures of the anion in 3.

Bond type	Measured bond length	Calculated bond length
Sn/Sb1–Sn/Sb2	3.169(3)	3.190
Sn/Sb3–Sn/Sb4	2.9864(10)	3.011
Sn/Sb3–Sn/Sb5	3.0832(11)	3.138
Sn/Sb4–Sn/Sb5	3.0685(10)	3.137
Sb/Sn1–Sn/Sb1	2.949(5)	2.987
Sb/Sn1–Sn/Sb2	2.911(2)	2.986
Sb/Sn1–Sn/Sb3	2.952(3)	2.968
Sb/Sn1–Sn/Sb4	2.942(3)	2.971
Sb/Sn2–Sn/Sb1	2.942(4)	2.924
Sb/Sn2–Sn/Sb3	2.8983(10)	2.938
Sb/Sn2–Sn/Sb5	2.9516(11)	2.972
Sb/Sn3–Sn/Sb2	2.8953(10)	2.924
Sb/Sn3–Sn/Sb4	2.9044(10)	2.939
Sb/Sn3–Sn/Sb5	2.9408(11)	2.972
Au1···Sn/Sb1	3.183(3)	3.182
Au1–Sn/Sb1^{i}	2.767(2)	2.808
Au1···Sn/Sb2	3.2248(8)	3.183
Au1–Sn/Sb2^{i}	2.7884(8)	2.809
Au1–Sb/Sn2	2.7607(8)	2.769
Au1–Sb/Sn3	2.7664(8)	2.771
Au1···Au1^{i}	2.8907(8)	2.886
Au1···Sn/Sb5	2.9407(8)	2.901

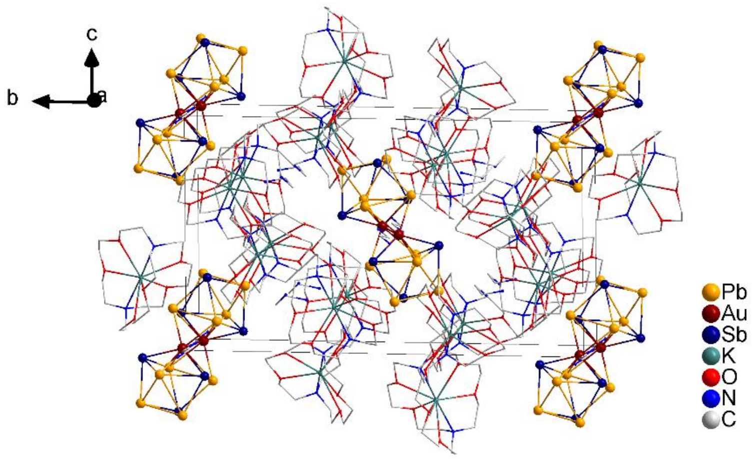

Figure S8. Unit cell of compound **4**.

Table S5. Selected interatomic distances (in Å) of the experimental and optimized structures of the anion in 4.

Bond type	Measured bond length	Calculated bond length
Pb1–Pb2	3.3114(7)	3.318
Pb3–Pb4	3.1444(7)	3.194
Pb3–Pb5	3.2285(7)	3.306
Pb4–Pb5	3.2268(7)	3.305
Sb1–Pb1	3.0573(8)	3.098
Sb1–Pb2	3.0703(13)	3.098
Sb1–Pb3	3.0101(9)	3.041
Sb1–Pb4	3.0213(8)	3.043
Sb2–Pb1	2.9571(9)	3.025
Sb2–Pb3	2.9912(9)	3.016
Sb2–Pb5	3.0028(9)	3.044
Sb3–Pb2	2.9817(8)	3.024
Sb3–Pb4	2.9811(12)	3.017
Sb3–Pb5	3.0035(10)	3.043
Au1···Pb1	3.2236(6)	3.178
Au1–Pb1^{i}	2.8951(12	2.926
Au1···Pb2	3.2418(8)	3.181
Au1–Pb2^{i}	2.8671(7)	2.926
Au1–Sb2	2.7475(10)	2.769
Au1–Sb3	2.7293(8)	2.772
Au1···Au1^{i}	2.8957(8)	2.870
Au1···Pb5	3.0528(10)	2.984

3. Energy Dispersive X-ray Spectroscopy (EDS) Analysis and Micro-X-ray Fluorescence Spectroscopy (µ-XFS) Analysis

3.1. Energy Dispersive X-ray Spectroscopy (EDS) of 1

EDS analysis of was carried out using an EDS-device Voyager 4.0 of Noran Instruments coupled with an electron microscope CamScan CS 4DV. Data acquisition was performed with an acceleration voltage of 25 kV and 100 s accumulation time. Results are summarized in Table S6 and illustrated in Figure S9. Note that the deviation of the K content is observed very frequently in EDS analyses of these very air-sensitive Zintl cluster compounds.

Table S6. EDS analysis of 1 (K, Au, Ge, As).

			**1**		
Element	Element wt %	Weight % err. (1 sigma)	Atom %	Element ratio observed	Element ratio calc
K-K	2.57	1.78	6.82	1.55	3.00
Au-L	49.97	1.38	26.32	6.00	6.00
Ge-K	25.82	1.09	36.89	8.41	9.00
As-K	21.64	0.10	29.97	6.83	7.00
Total	100.00		100.00		

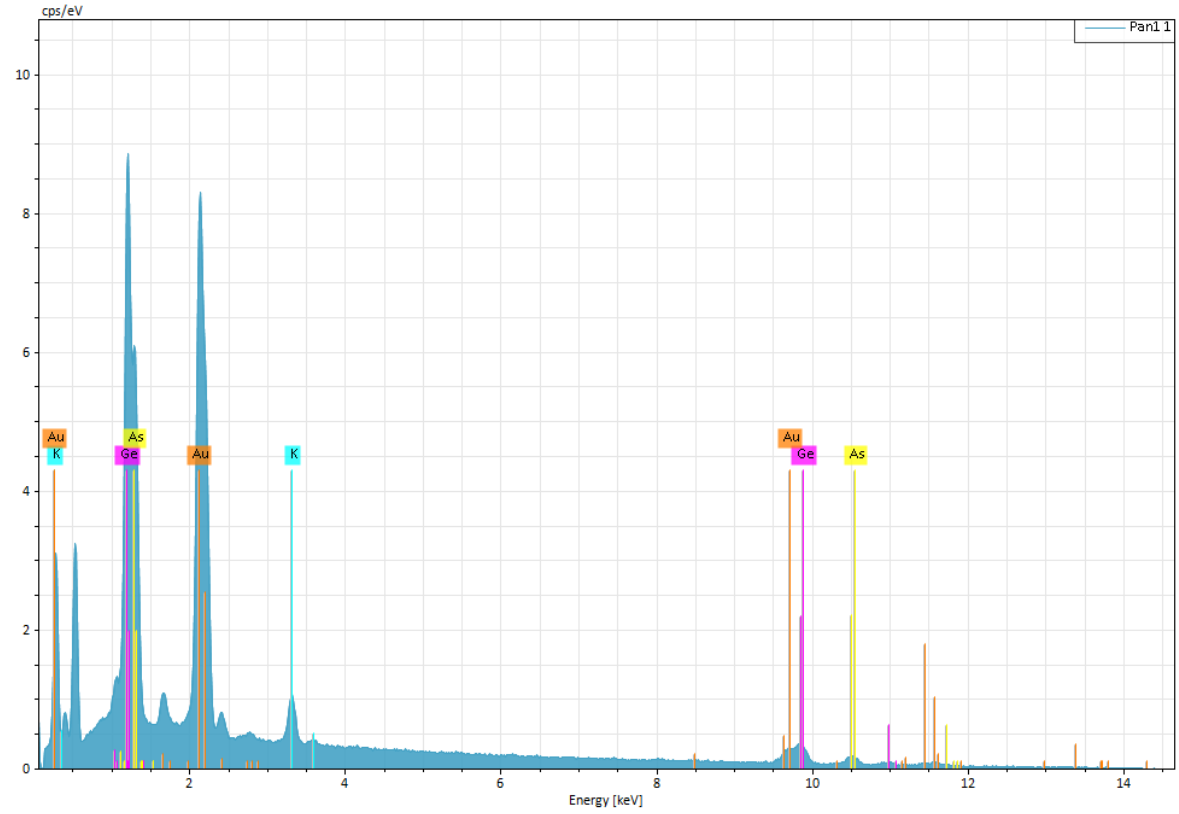

Figure S9. EDS analysis of **1**.

3.2. Micro-X-ray Fluorescence Spectroscopy (µ-XFS) Analysis

All µ-XFS measurements were performed with a Bruker M4 Tornado, equipped with an Rh-target X-ray tube and a Si drift detector. The emitted fluorescence photons are detected with an acquisition time of 100 s. Quantification of the elements is achieved through deconvolution of the spectra. Results are summarized in Table S7. Figures S10-S12 present the spectra for **2**–**4** along with the results of the deconvolution algorithm. Several measurements produced unreasonably large values for the % K. Removal of K from the calculations afforded excellent

agreement with the expected atomic ratio of close to $Au_{2.00}Sn_{10.00}Pn_{6.00}$ in **2**–**4**. We assume that accumulation of K at the crystal surface upon exposure to air is responsible for the anomalous results. This is observed commonly for very these very air-sensitive compounds.

Table S7. μ-XFS analysis of 2 - 4 (K, Au, Sn, Pb, Sb, Bi).

			**2**		
Element	Element wt %	Weight % err. (1 sigma)	Atom %	Element ratio observed	Element ratio calc
K-K	15.11	0.00	41.36	14.11	4.00
Au-L	10.78	0.00	5.86	2.00	2.00
Sn-L	38.01	0.00	34.28	11.70	10.00
Bi-L	36.11	0.00	18.50	6.31	6.00
Total	100.00		100.00		
			**3**		
Element	Element wt %	Weight % err. (1 sigma)	Atom %	Element ratio observed	Element ratio calc
K-K	11.55	0.00	30.01	7.80	4.00
Au-L	14.90	0.00	7.69	2.00	2.00
Sn-L	41.96	0.00	35.93	9.34	10.00
Sb-L	31.59	0.00	26.37	6.86	6.00
Total	100.00		100.00		
			**4**		
Element	Element wt %	Weight % err. (1 sigma)	Atom %	Element ratio observed	Element ratio calc
K-K	4.10	0.00	16.22	3.32	4.00
Au-L	12.44	0.00	9.77	2.00	2.00
Pb-L	61.20	0.00	45.71	9.36	10.00
Sb-L	22.27	0.00	28.30	5.79	6.00
Total	100.00		100.00		

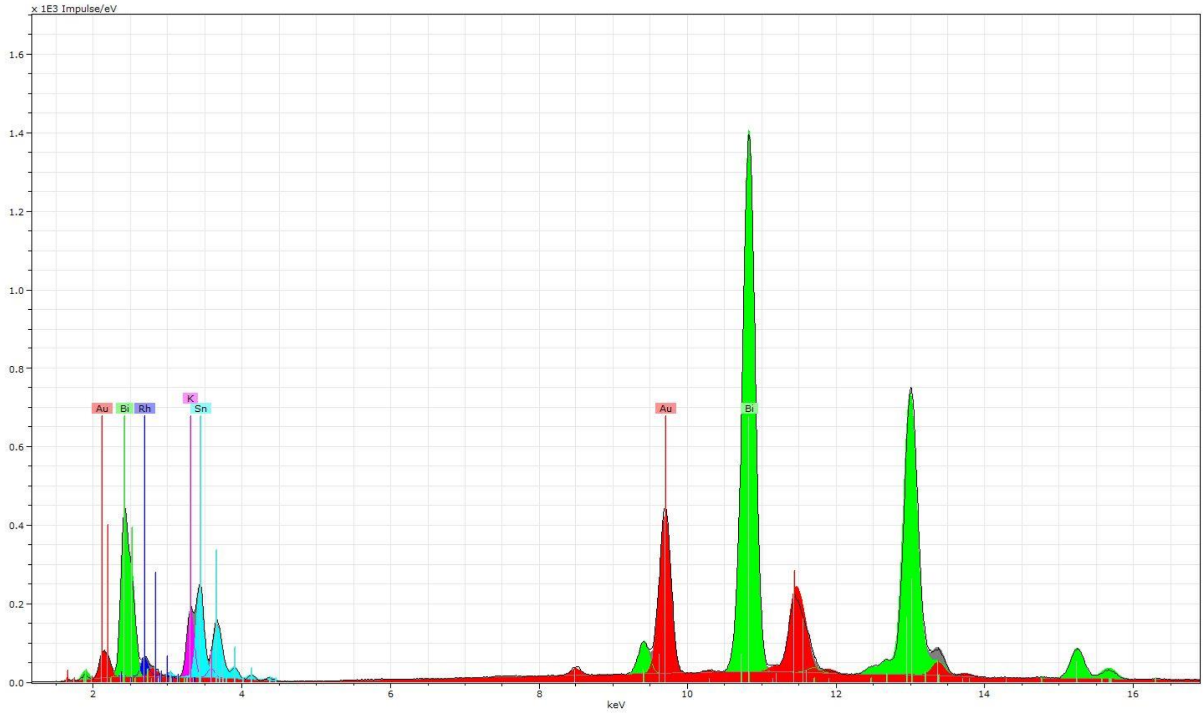

Figure S10. Micro X-ray fluorescence spectrum of **2** with the results of the deconvolution algorithm. Colors are used as follows: K (pink), Au (red), Sn (light blue), Bi (green).

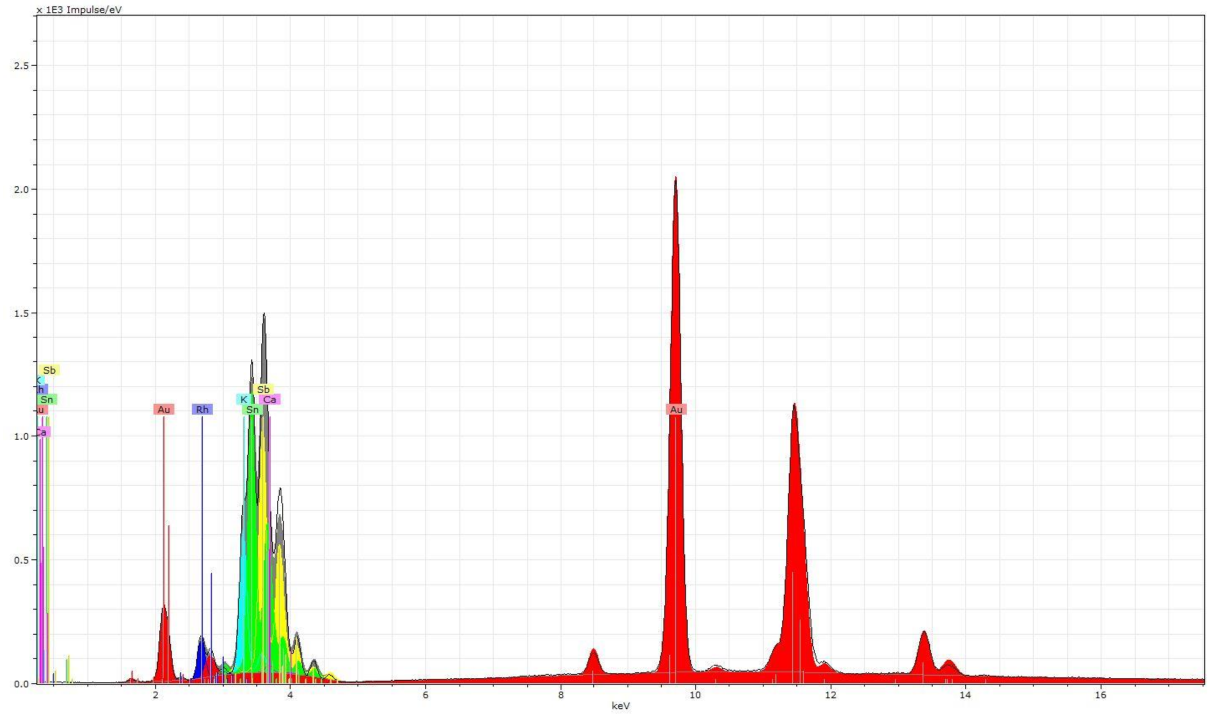

Figure S11. Micro X-ray fluorescence spectrum of compound **3** with the results of the deconvolution algorithm. Colors are used as follows: K (light blue), Au (red), Sn (green), Sb (yellow).

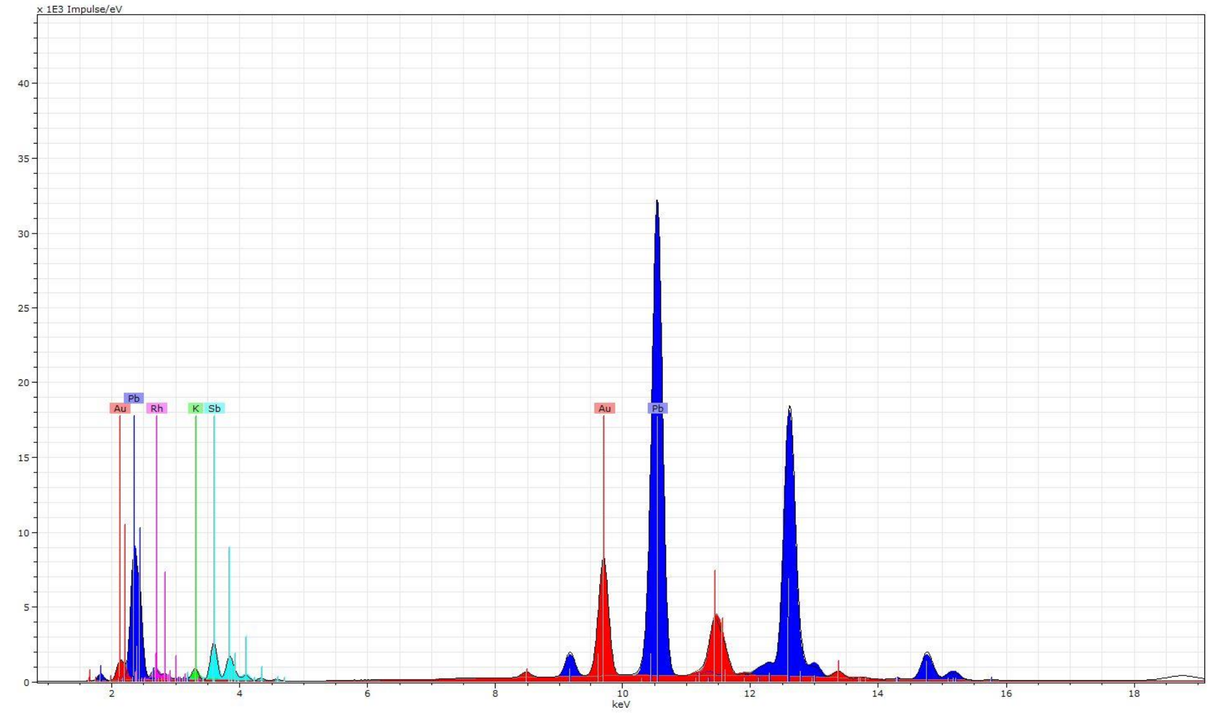

Figure S12. Micro X-ray fluorescence spectrum of compound **4** with the results of the deconvolution algorithm. Colors are used as follows: K (green), Au (red), Pb (blue), Sb (light blue).

4. Electrospray ionization mass spectrometry (ESI-MS) Investigations

4.1. Methods

All mass spectra were recorded with a Thermo Fischer Scientific Finnigan LTQ-FT spectrometer in negative ion mode. We prepared a fresh reaction solution of the reaction leading to compound **1** in DMF, and solutions of single crystals of the compounds **2**–**4** in freshly distilled DMF inside a glovebox. The solutions were injected into the spectrometer with gastight 250 µL Hamilton syringes by syringe pump infusion. All capillaries within the system were washed with dry DMF for 2 hours before and at least 10 minutes in between measurements to avoid decomposition reactions and consequent clogging.

The following ESI parameters were used: Spray Voltage: 3.6 kV, Capillary Temp: 290 °C, Capillary Voltage: −20, Tube lens Voltage: −121.75, Sheath Gas: 45, Sweep Gas: 0, Auxiliary Gas: 40. Assignable high-resolution mass peaks are shown in Figures S13-S31.

4.2. Mass spectra of the reaction solution yielding 1

ESI-MS measurements on single crystals of **1** failed, which we attribute to its larger molar mass in combination with a rather coordination-type bonding. Yet, we were able to identify important species in the reaction solution, which indicate the co-existence of $(Ge_2As_2)^{2-}$ (in protonated, monoanionic form), $(Ge_3As)^{3-}$, and also $(GeAs_3)^{-}$. The latter is not part of the cluster anion, yet must form alongside of $(Ge_3As)^{3-}$, according to equation (1) provided in the main document. Hence, we can take the spectra as further indication of the correctness of our findings. The high resolution images of the said species are provided in Figures S13-S17. Note that ESI mass spectra of Zintl anions always show monoanions, even though the original charge was higher.

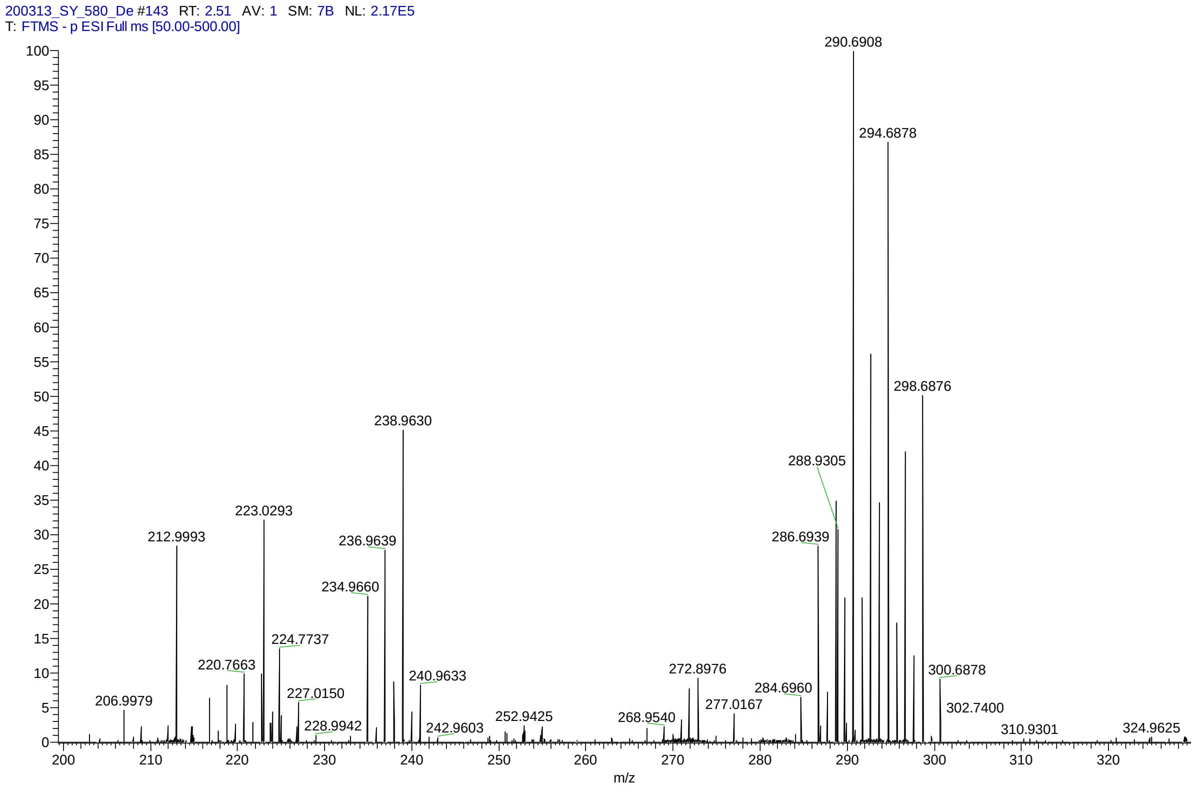

Figure S13. Overview of ESI(−) mass spectrum recorded immediately upon injection of a fresh reaction solution of **1** in DMF.

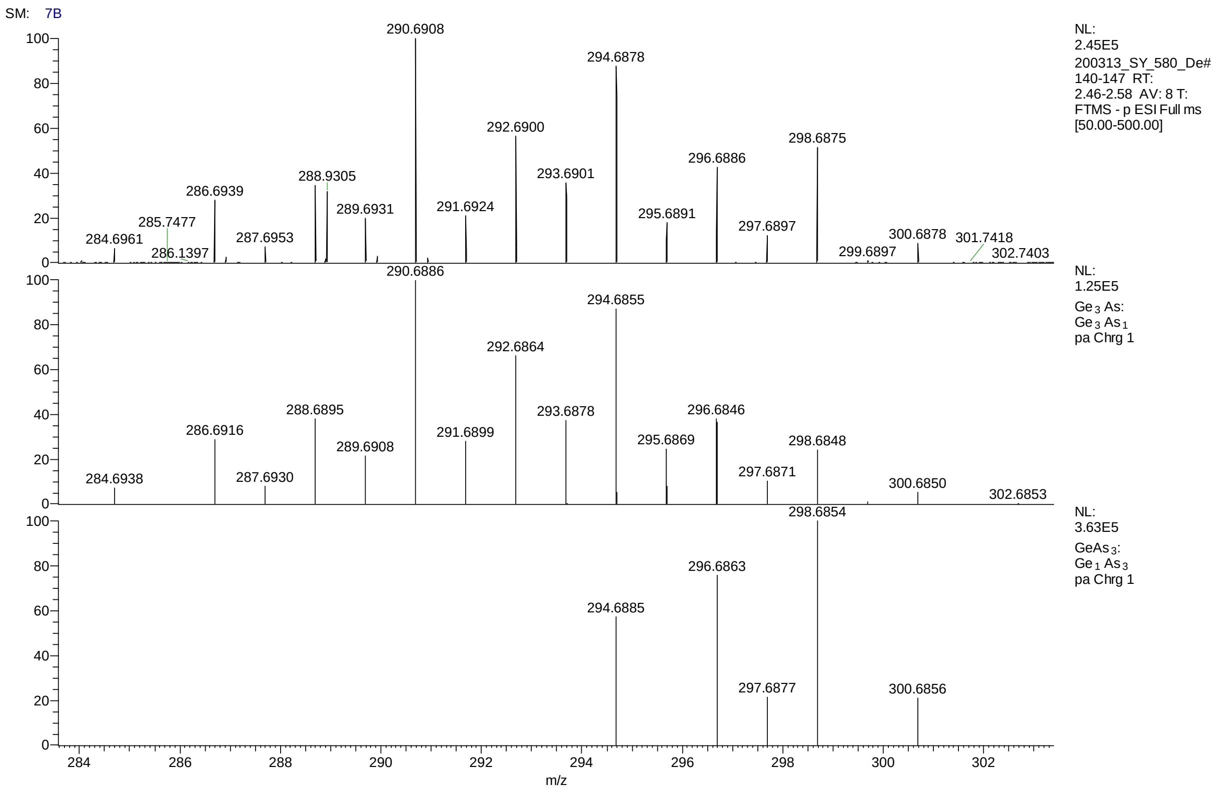

Figure S14. High-resolution ESI mass spectrum in negative ion mode recorded immediately upon injection of a fresh reaction solution of **1** in DMF, indicating the coexistence of $(Ge_3As)^-$ and $(GeAs_3)^-$. Topmost: measured, below: simulated.

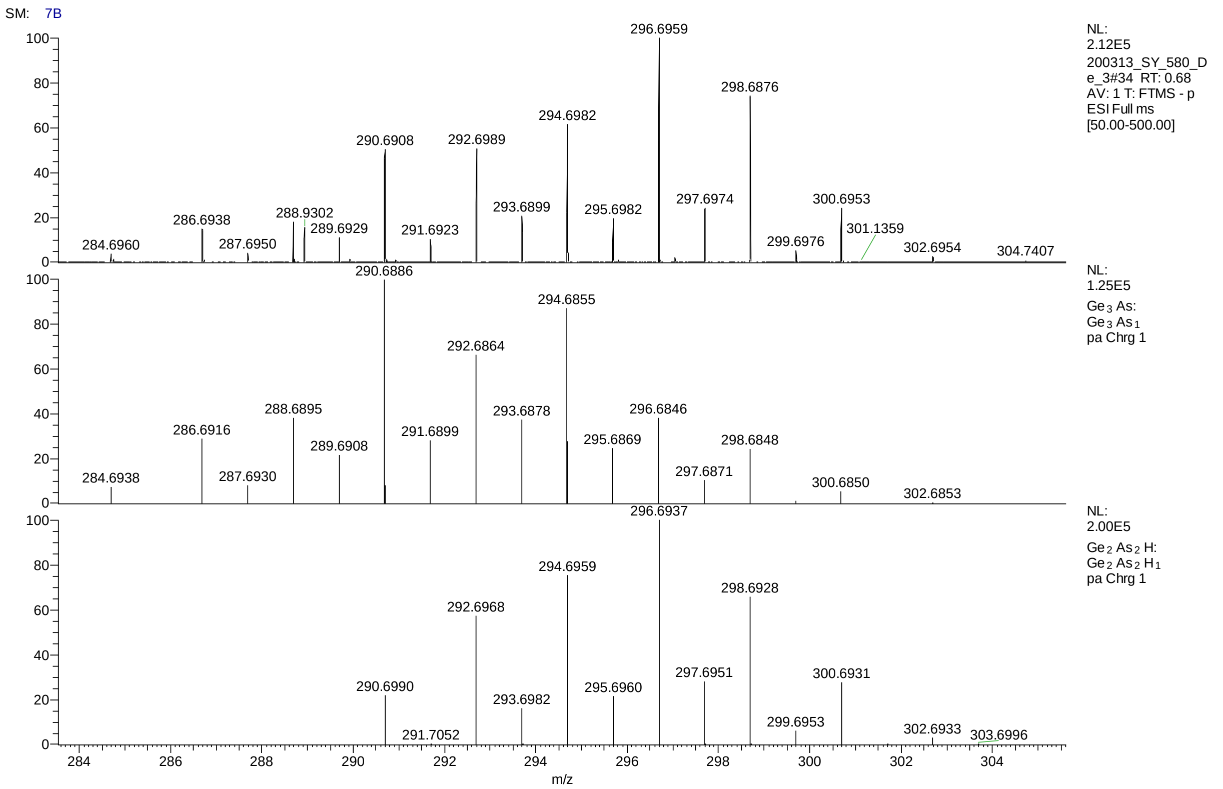

Figure S15. High-resolution ESI mass spectrum in negative ion mode recorded immediately upon injection of a fresh reaction solution of **1** in DMF, indicating the coexistence of $(Ge_3As)^-$ and $(Ge_2As_2H)^-$. Topmost: measured, below: simulated.

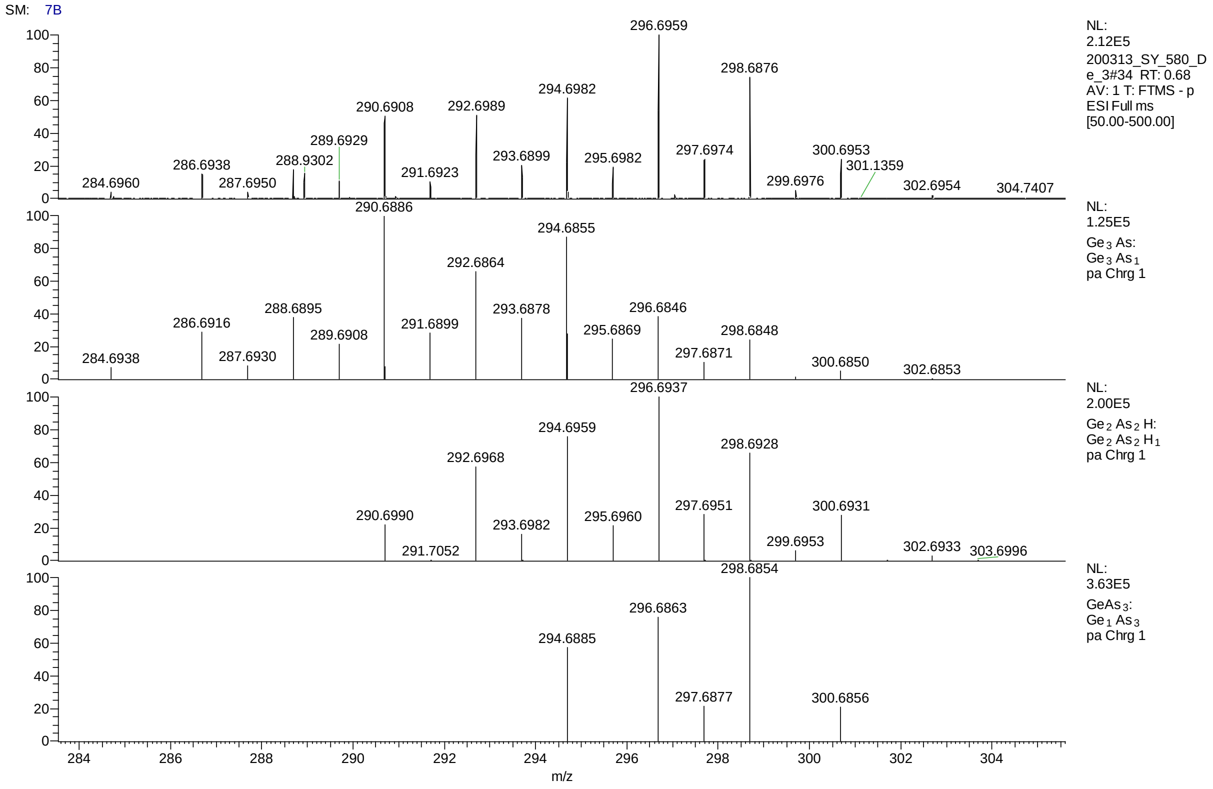

Figure S16. High-resolution ESI mass spectrum in negative ion mode recorded immediately upon injection of a fresh reaction solution of **1** in DMF, indicating the coexistence of $(Ge_3As)^-$ and $(Ge_2As_2H)^-$, and $(GeAs_3)^-$. Topmost: measured, below: simulated.

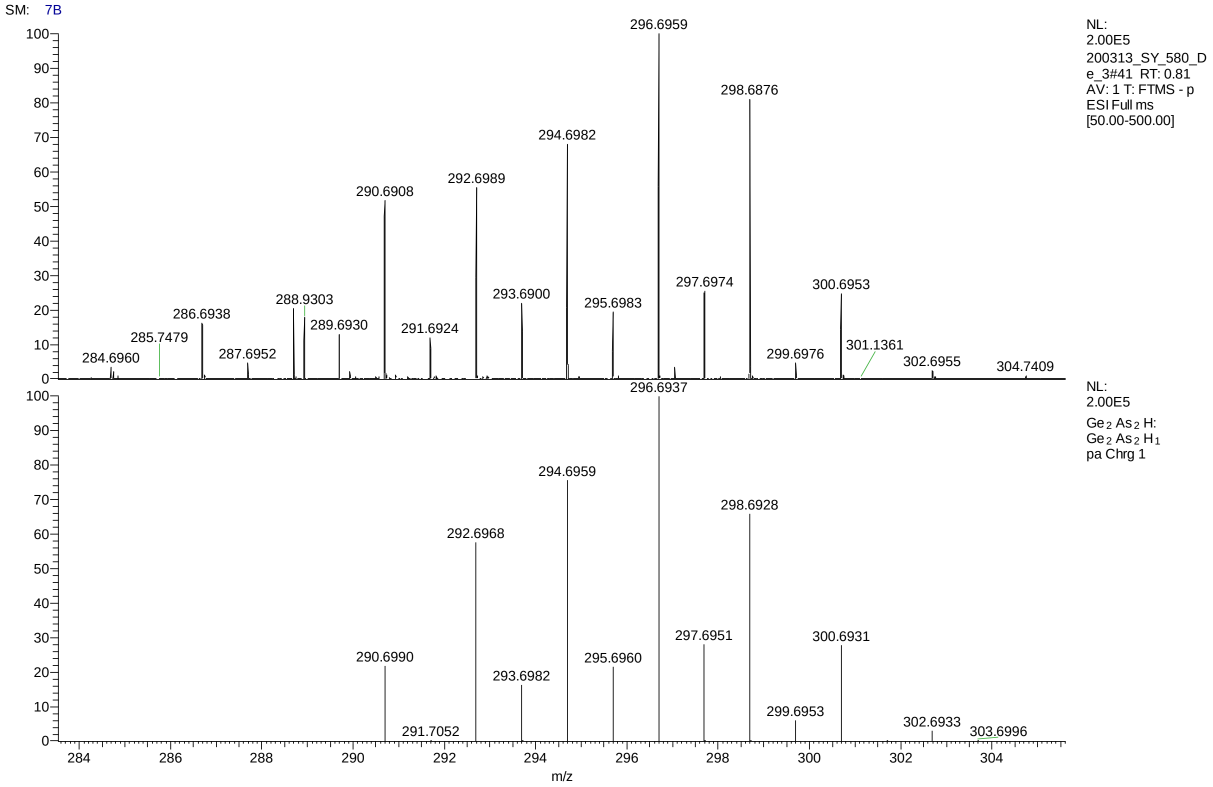

Figure S17. High-resolution ESI mass spectrum in negative ion mode recorded immediately upon injection of a fresh reaction solution of **1** in DMF, indicating the occurrence of $(Ge_2As_2H)^-$. Top: measured, bottom: simulated.

4.3. Mass spectra of 2

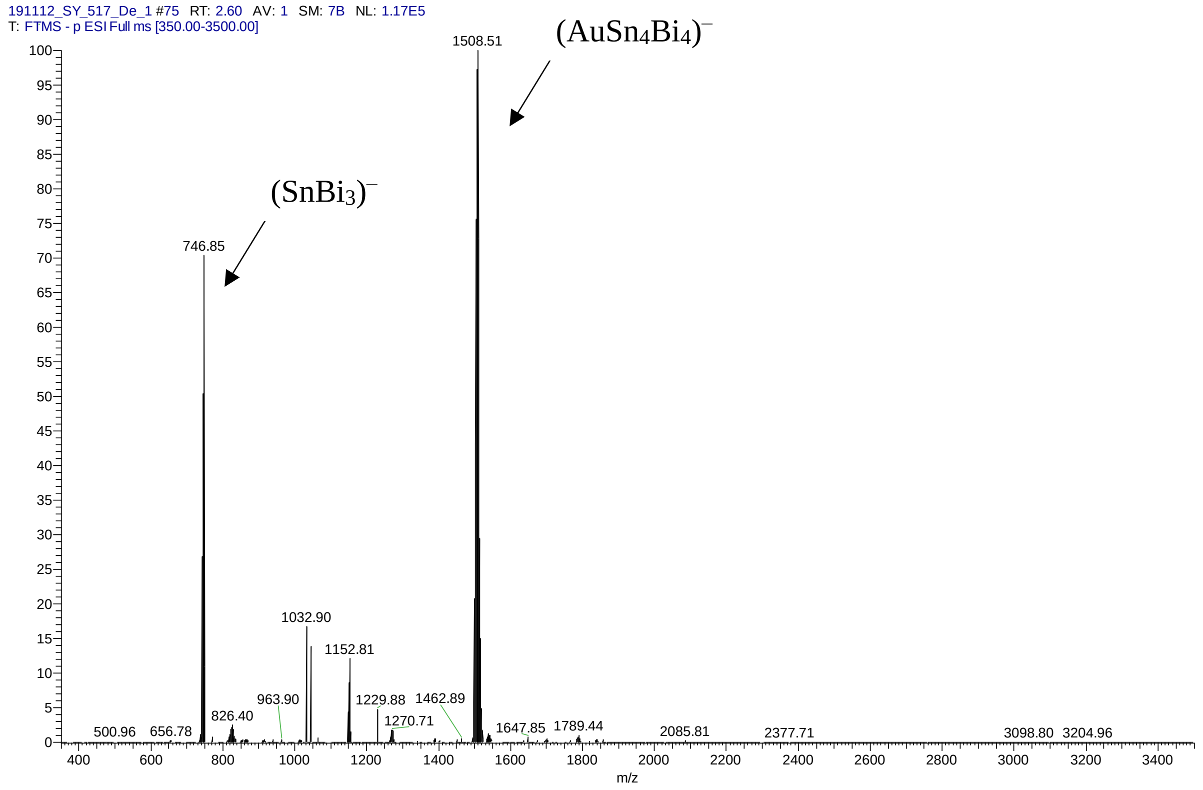

Figure S18. Overview of ESI(−) mass spectrum recorded immediately upon injection of a fresh solution of **2** in DMF.

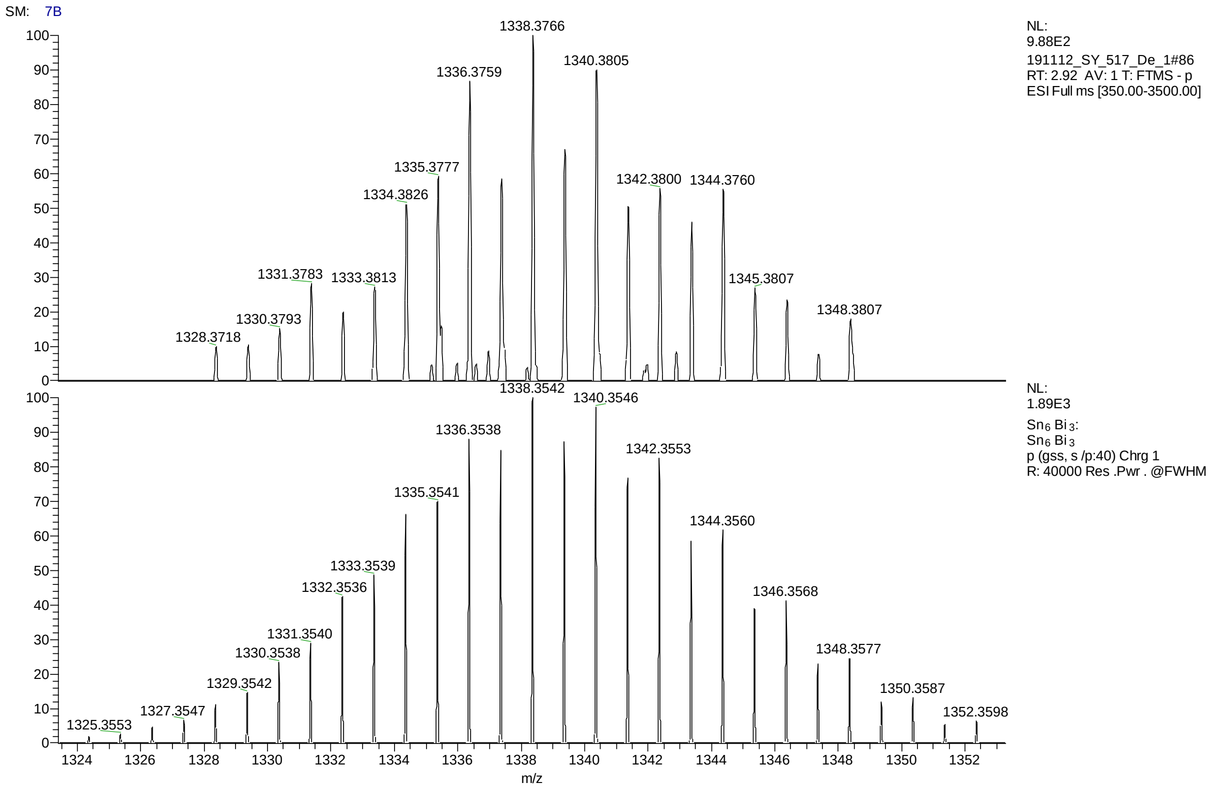

Figure S19. High-resolution ESI mass spectrum in negative ion mode of $(Sn_6Bi_3)^-$, recorded immediately upon injection of a fresh solution of **2** in DMF. Top: measured, bottom: simulated.

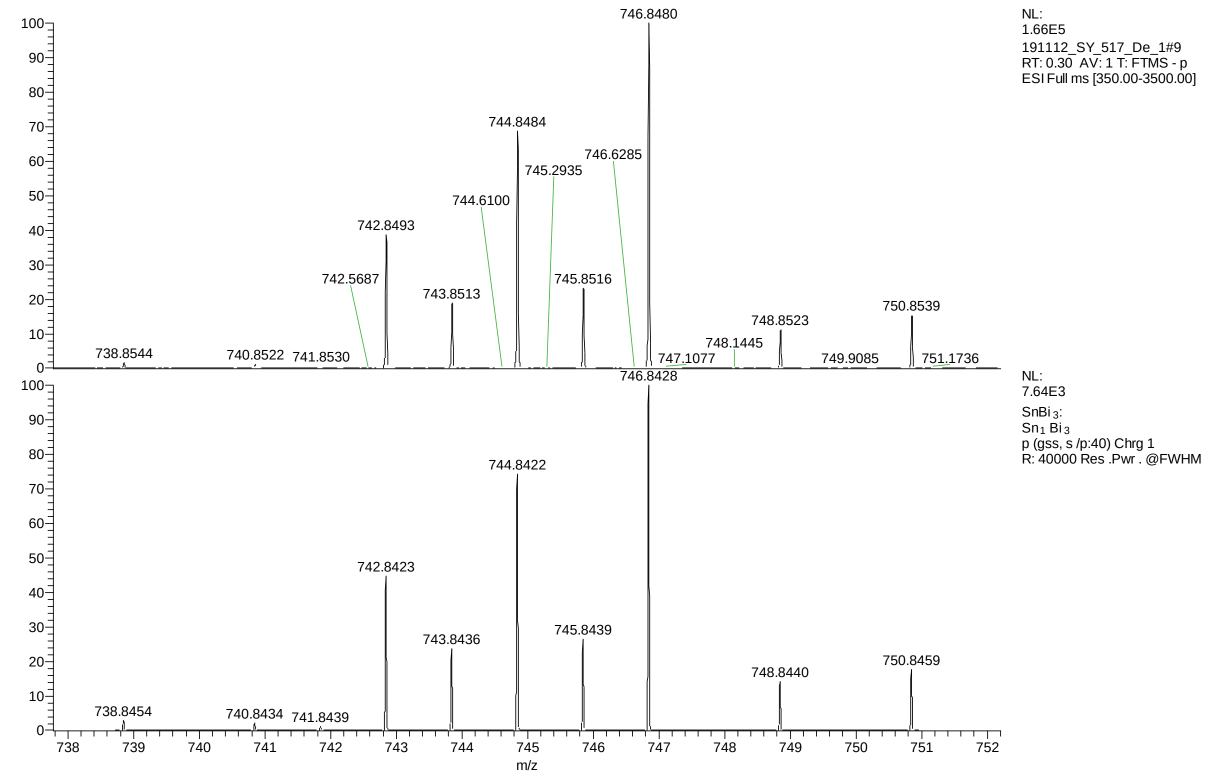

Figure S20. High-resolution ESI mass spectrum in negative ion mode of $(SnBi_3)^-$, recorded immediately upon injection of a fresh solution of **2** in DMF. Top: measured, bottom: simulated.

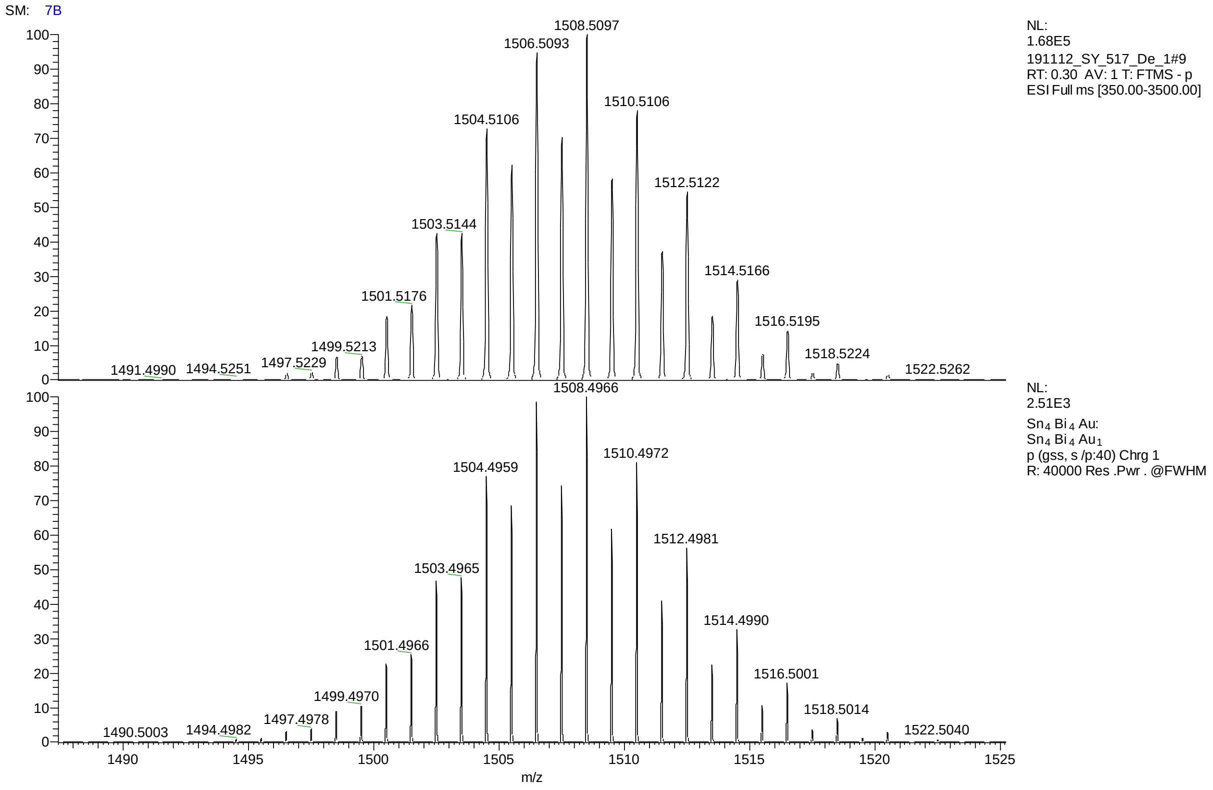

Figure S21. High-resolution ESI mass spectrum in negative ion mode of $(AuSn_4Bi_4)^-$, recorded immediately upon injection of a fresh solution of **2** in DMF. Top: measured, bottom: simulated.

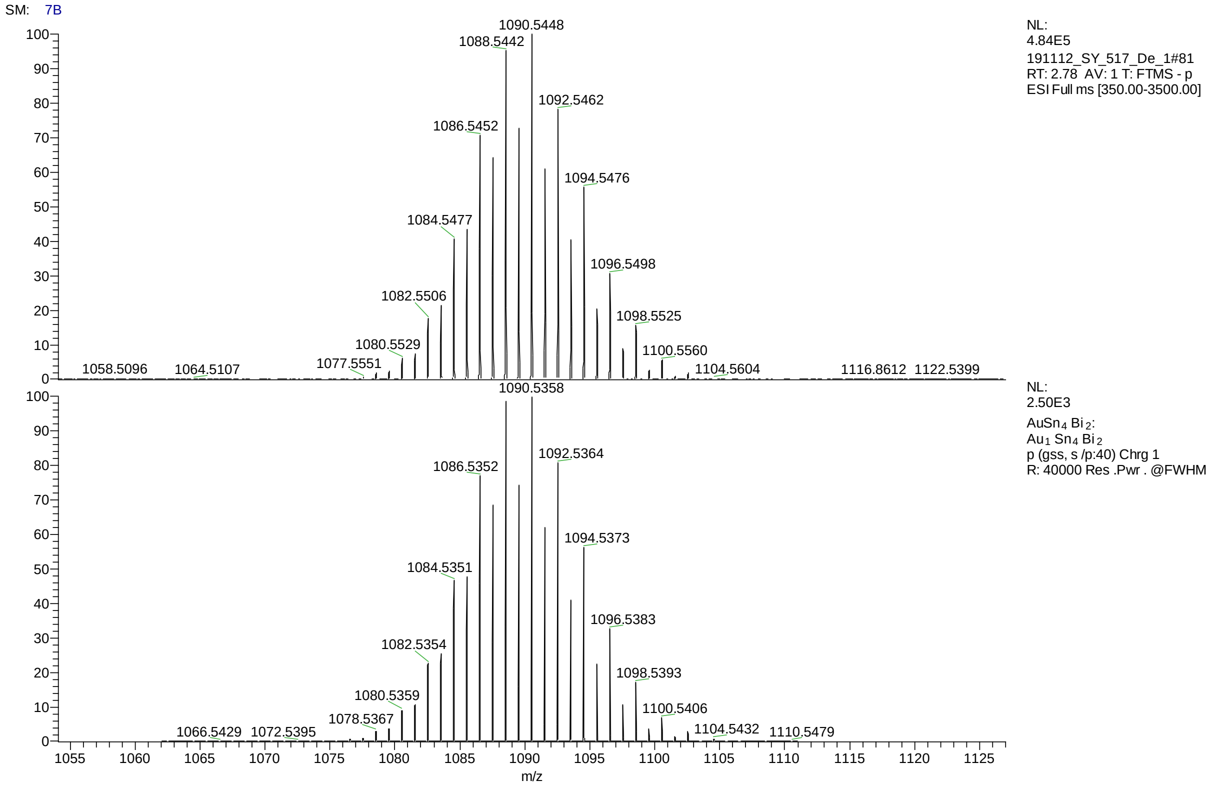

Figure S22. High-resolution ESI mass spectrum in negative ion mode of $(AuSn_4Bi_2)^-$, recorded immediately upon injection of a fresh solution of **2** in DMF. Top: measured, bottom: simulated.

4.4. Mass spectra of 3

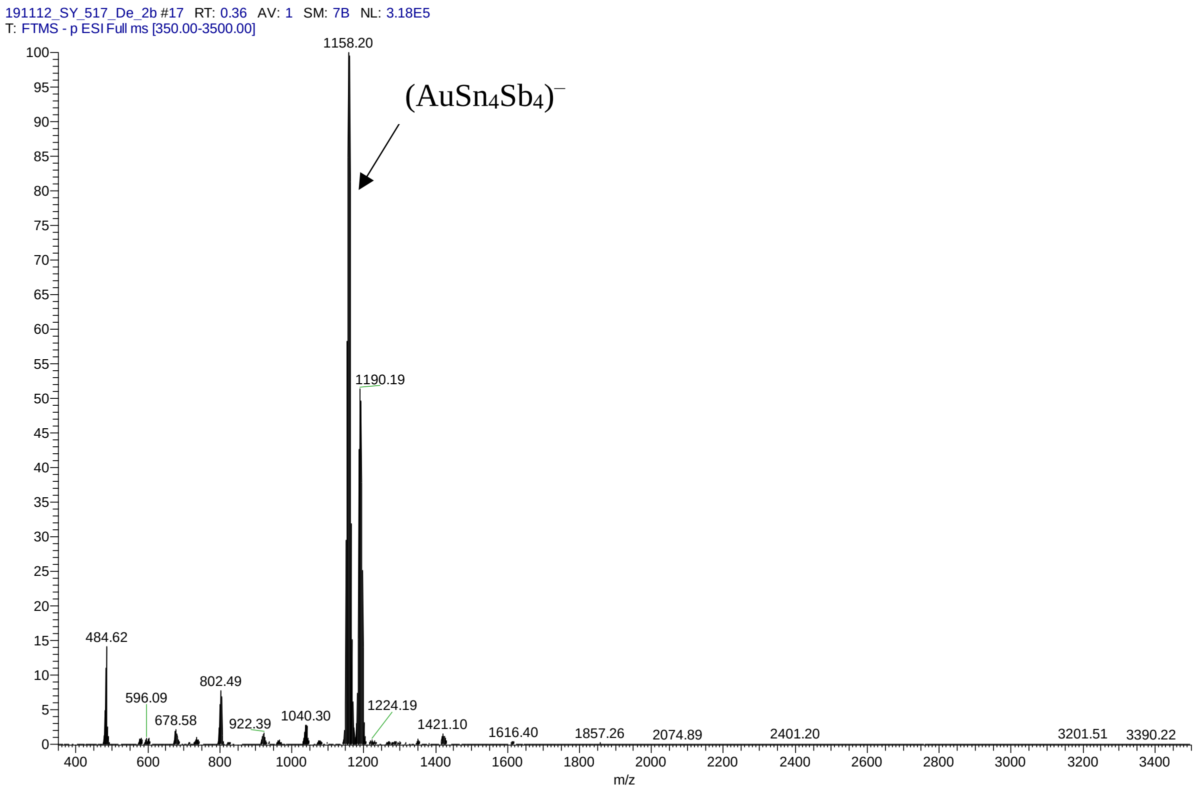

Figure S23. Overview of ESI(−) mass spectrum recorded immediately upon injection of a fresh solution of **3** in DMF.

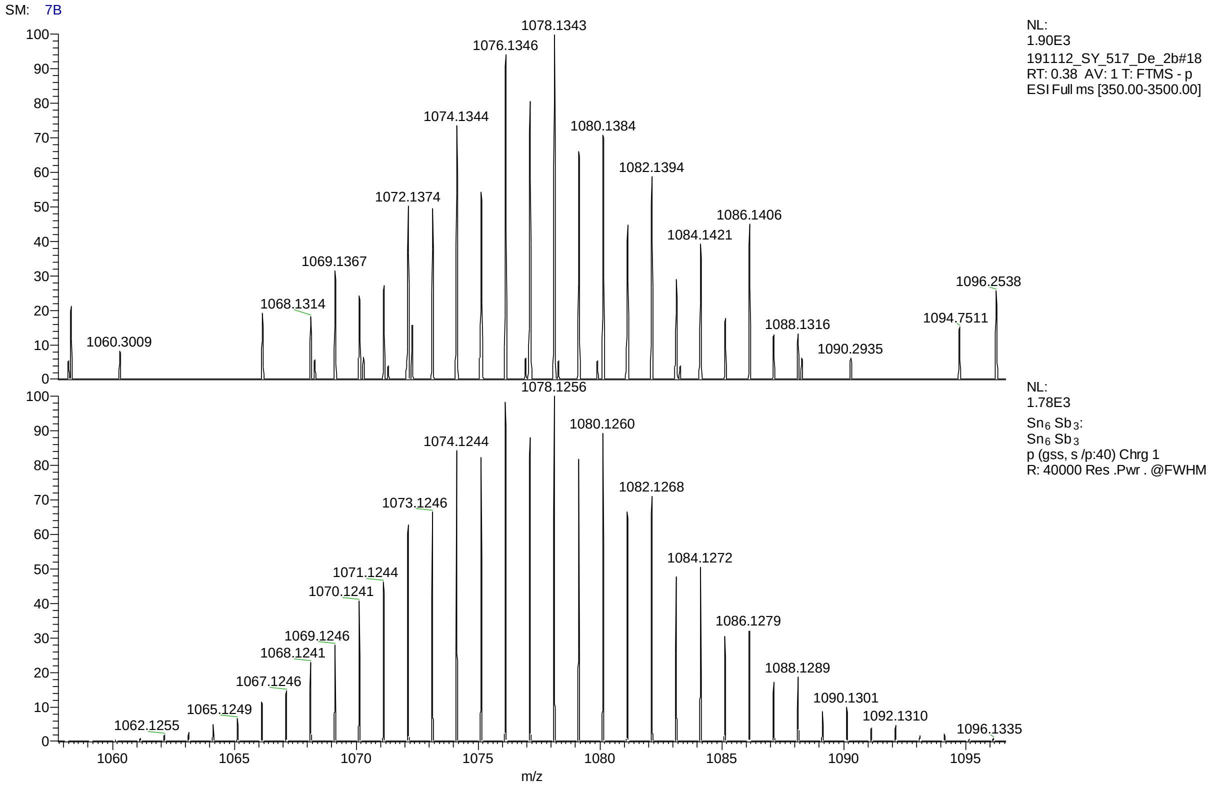

Figure S24. High-resolution ESI mass spectrum in negative ion mode of $(Sn_6Sb_3)^-$, recorded immediately upon injection of a fresh solution of **3** in DMF. Top: measured, bottom: simulated.

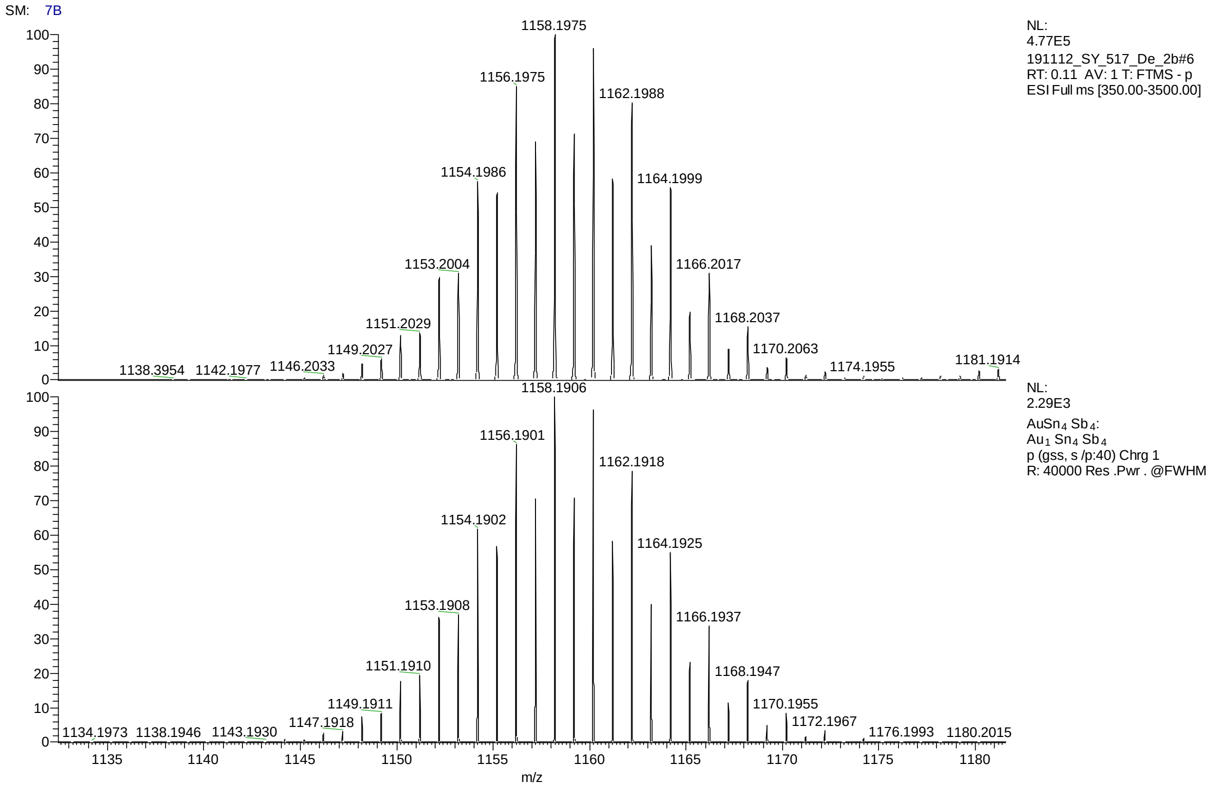

Figure S25. High-resolution ESI mass spectrum in negative ion mode of $(AuSn_4Sb_4)^-$, recorded immediately upon injection of a fresh solution of **3** in DMF. Top: measured, bottom: simulated.

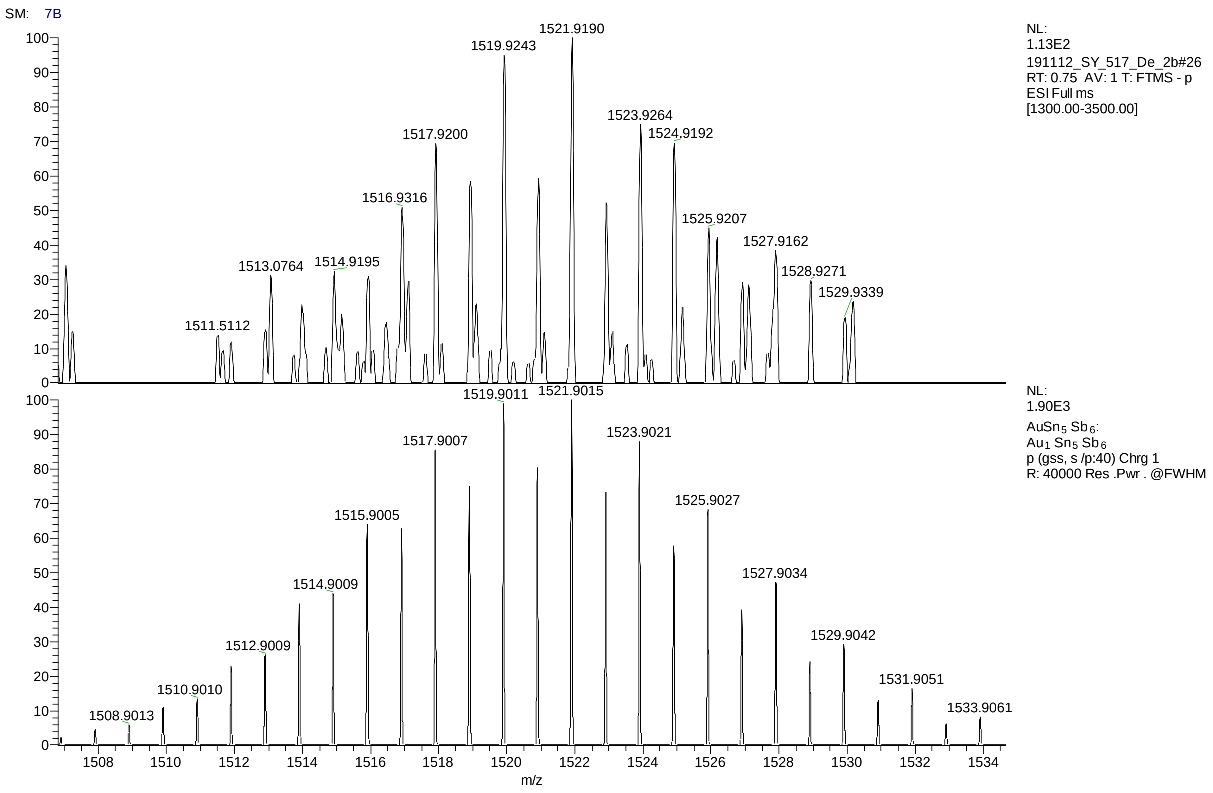

Figure S26. High-resolution ESI mass spectrum in negative ion mode of $(AuSn_5Sb_6)^-$, recorded immediately upon injection of a fresh solution of **3** in DMF. Top: measured, bottom: simulated.

4.5. Mass spectra of 4

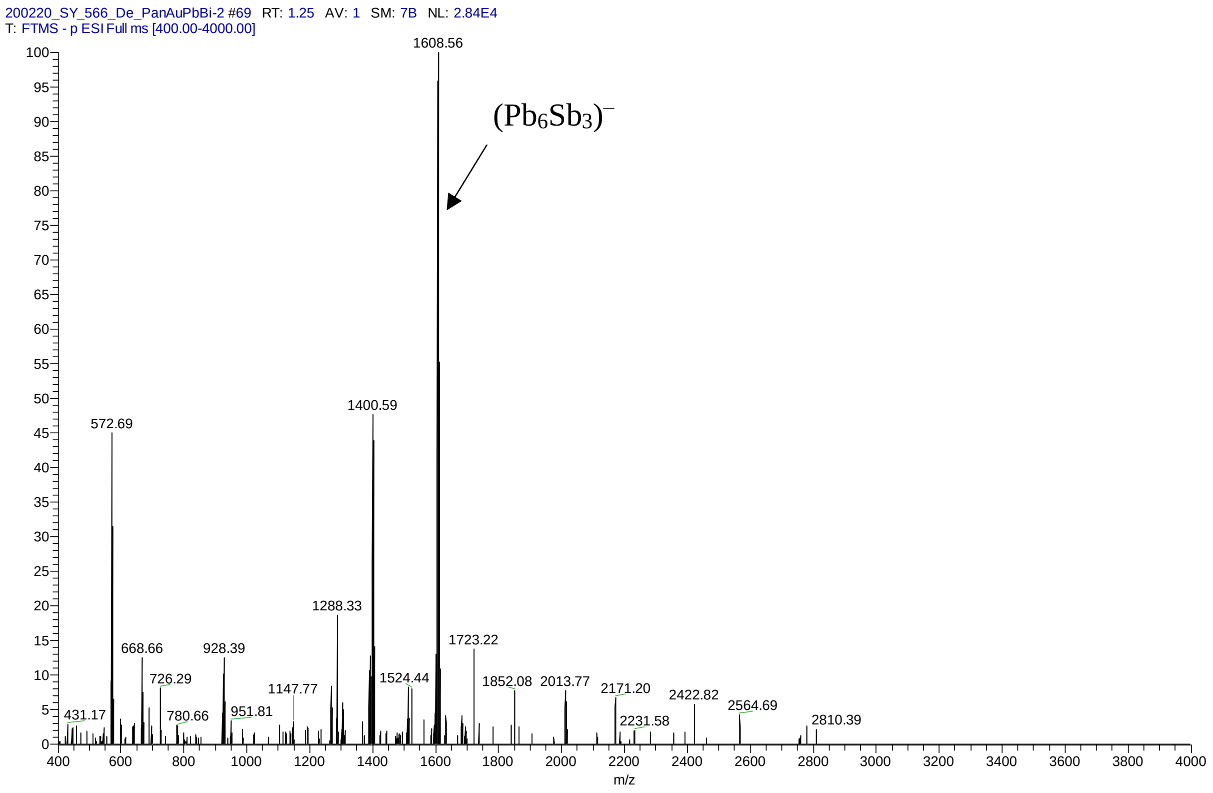

Figure S27. Overview of ESI(−) mass spectrum recorded immediately upon injection of a fresh solution of **4** in DMF.

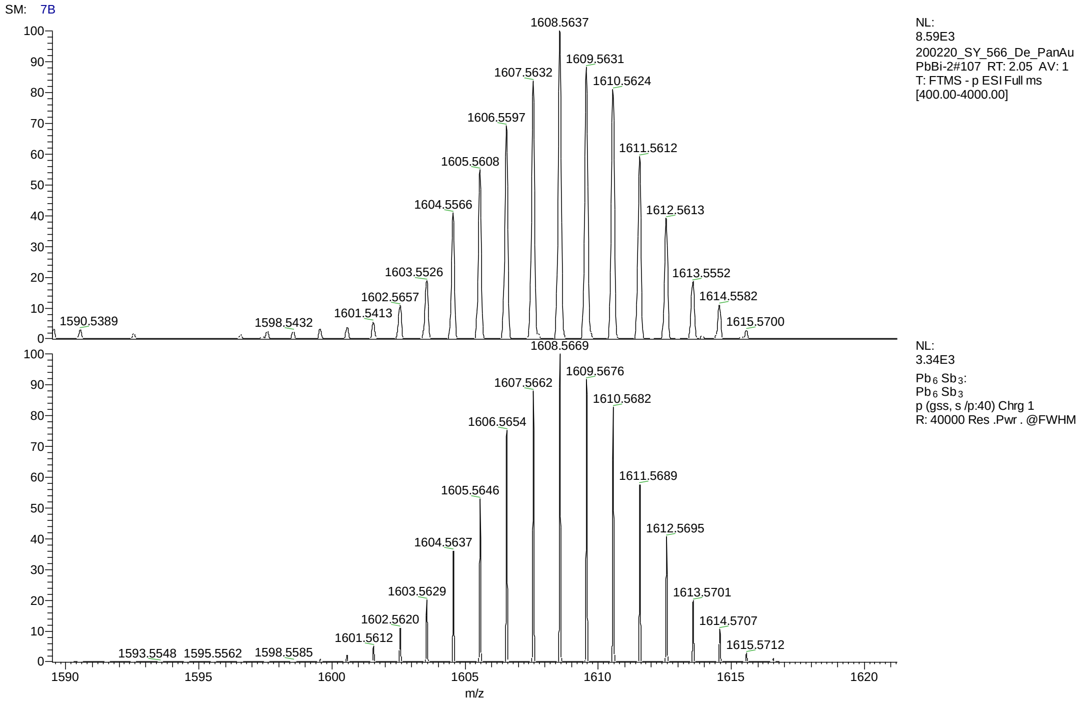

Figure S28. High-resolution ESI mass spectrum in negative ion mode of $(Pb_6Sb_3)^-$, recorded immediately upon injection of a fresh solution of **3** in DMF. Top: measured, bottom: simulated.

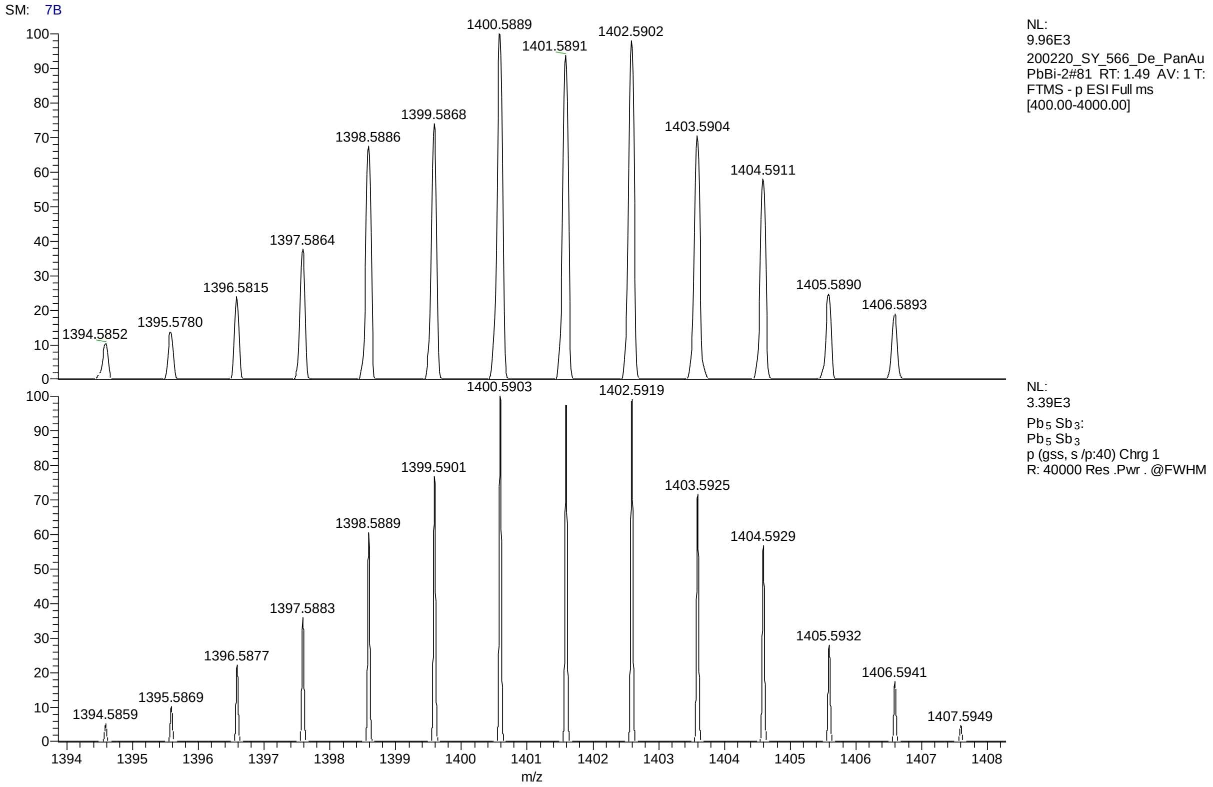

Figure S29. High-resolution ESI mass spectrum in negative ion mode of $(Pb_5Sb_3)^-$, recorded immediately upon injection of a fresh solution of **3** in DMF. Top: measured, bottom: simulated.

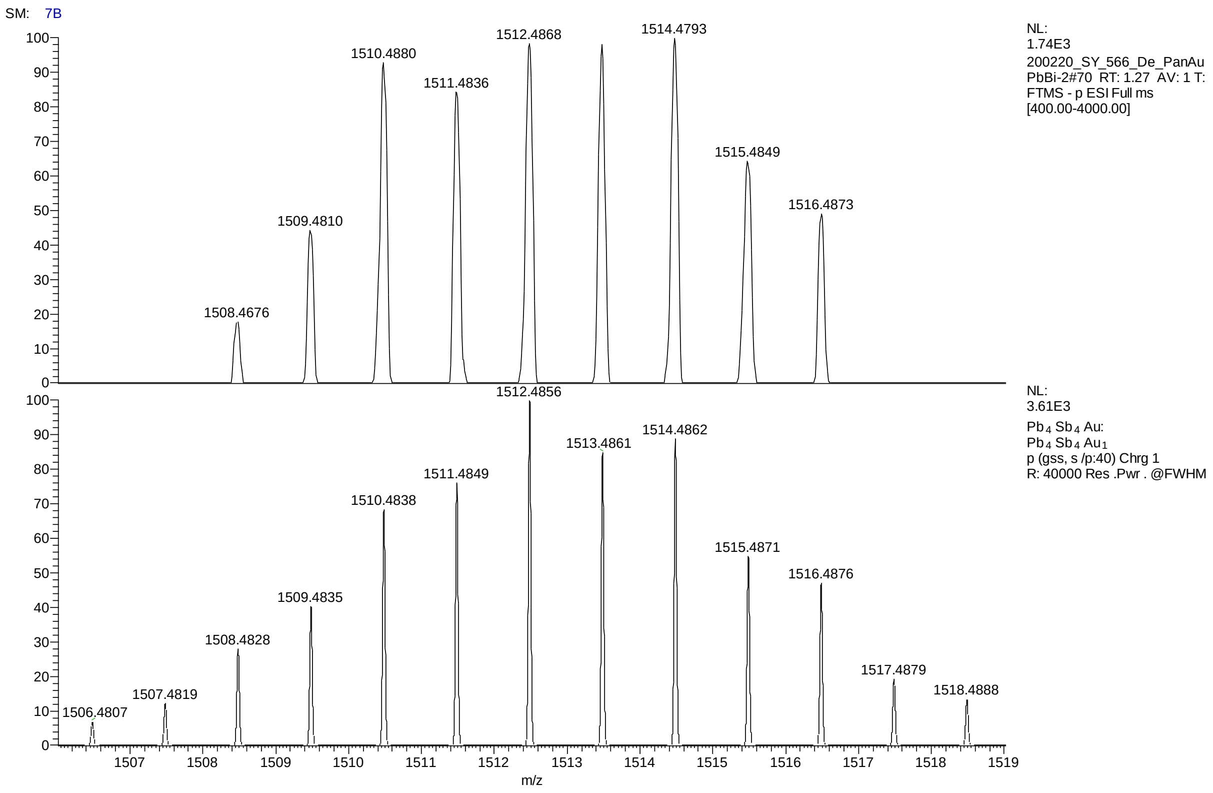

Figure S30. High-resolution ESI mass spectrum in negative ion mode of $(AuPb_4Sb_4)^-$, recorded immediately upon injection of a fresh solution of **3** in DMF. Top: measured, bottom: simulated.

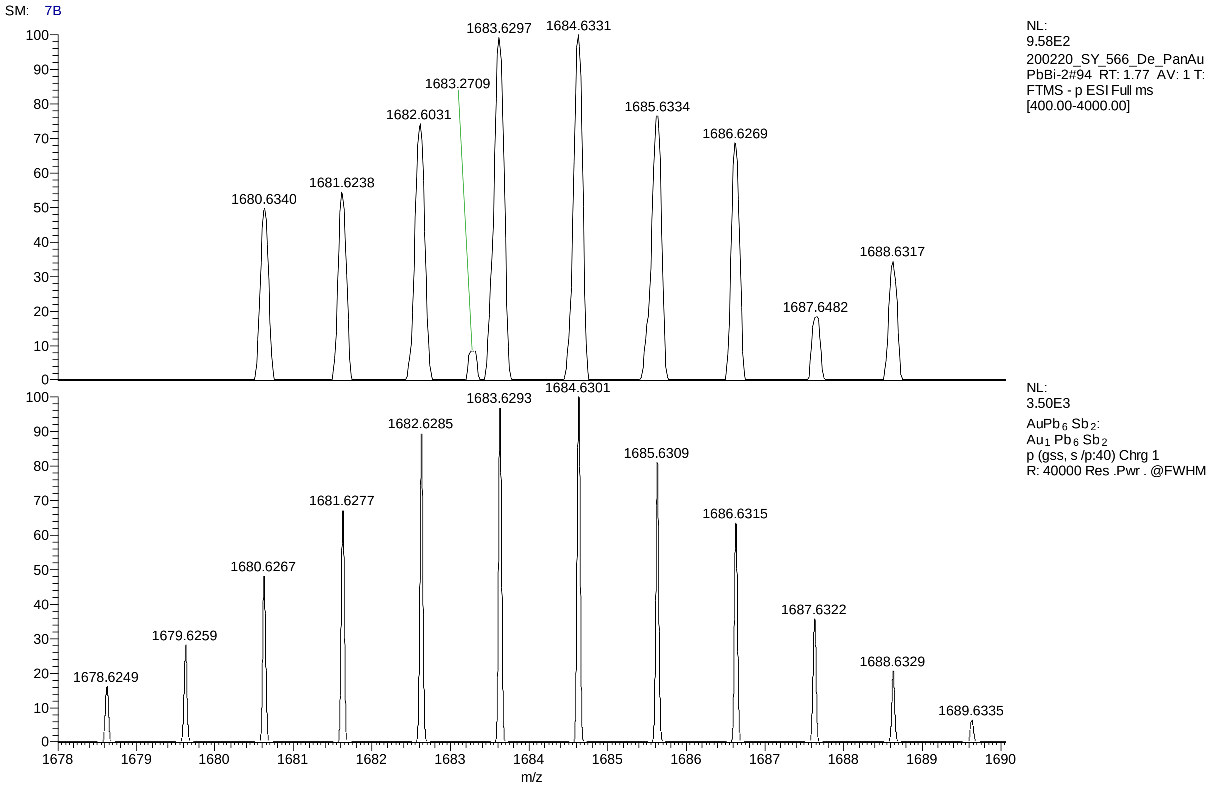

Figure S31. High-resolution ESI mass spectrum in negative ion mode of $(AuPb_6Sb_2)^-$, recorded immediately upon injection of a fresh solution of **3** in DMF. Top: measured, bottom: simulated.

5. Quantum Chemical Investigations

5.1. Methods

All calculations were undertaken by employing density functional theory (DFT) methods as implemented in the program system TURBOMOLE V7.1.1.[6,7] We applied the TPSS functional.[8] The used basis sets were of the quality def2-TZVP (for the exchange reactions) and dhf-TZVP (for the study of the four dimeric anions $[(AuPb_5Bi_3)_2]^{4-}$ and **2**–**4**).[9] We additionally used the corresponding auxiliary basis sets[10] and effective core potentials at Sn, Pb, Sb, Bi, and Au atoms.[11]

The bonding situation – in particular that of the Au atoms − was examined by means of population analyses based on occupation numbers (Paboon)[12] and upon inspection of localized molecular orbitals (LMOs).[13] The pictures of the LMOs were created with gOpenMol.[14]

5.2 Reaction Energies for the Exchange Reactions

Table S8. Reaction energies for the exchange reactions, E_{XR}, corresponding to the first step of equation (1) in the main document.[a] Tt indicates the involved tetrel atom, Pn indicates the involved pnictogen atom.

Tt	Pn	E_{XR} / kJ mol^{-1}
Si	P	−66.16
	As	−61.03
	Sb	−51.92
	Bi	−34.99
Ge	P	−111.43
	As	−110.23
	Sb	−107.17
	Bi	−91.00
Sn	P	−40.90
	As	−50.20
	Sb	−59.44
	Bi	−49.24
Pn	P	−30.26
	As	−41.62
	Ab	−57.38
	Bi	−49.27

[a] $2\ (Tt_2Pn_2)^{2-} \rightleftharpoons (Tt_3Pn)^{3-} + (TtPn_3)^{-}$

Table S9. Geometrical consideration of *pseudo*-tetrahedral anions $(Tt_xPn_{4-x})^{2-}$. A denotes the atomic radii (in Å)[15] of the Tt atoms: 1.11 (Si), 1.20 (Ge), 1.39 (Sn), 1.46 (Pb). B denotes the atomic radii (in Å [15] of the Pn atoms: 1.07 (P), 1.19 (As), 1.39 (Sb), 1.48 (Bi).

Tt/Pn	(|B−A|)/(B+A)	(B-A)/(B+A)
Si/P	0,018	−0,018
Si/As	0,035	0,035
Si/Sb	0,112	0,112
Si/Bi	0,143	0,143
Ge/P	0,057	−0,057
Ge/As	0,004	−0,004
Ge/Sb	0,073	0,073
Ge/Bi	0,104	0,104
Sn/P	0,130	−0,130
Sn/As	0,076	−0,076
Sn/Sb	0,000	0,000
Sn/Bi	0,031	0,031
Pb/P	0,154	−0,154
Pb/As	0,102	−0,102
Pb/Sb	0,025	−0,025
Pb/Bi	0,007	0,007

5.3 Calculated Minimum Structure of $\{[AuPb_5Bi_3]_2\}^{4-}$

Figure S32 shows the calculated minimum structure of $\{[AuPb_5Bi_3]_2\}^{4-}$ and gives the corresponding structural data. The atom labelling corresponds to Figure 5 in the manuscript.

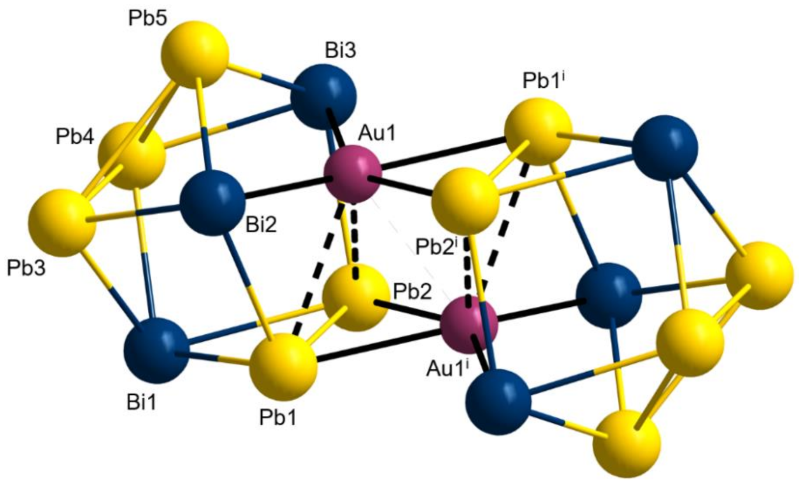

Figure S32. Calculated minimum structure for $\{[AuPb_5Bi_3]_2\}^{4-}$. Selected distances are given in Å: Pb1–Pb2 3.29, Pb3–Pb4 3.15, Pb(3,4)–Pb5 3.28, Bi1–Pb(1,2) 3.18, Bi1–Pb(3,4) 3.11, Bi(2,3)–Pb(1,2) 3.11, Bi(2,3)–Pb(3,4) 3.10, Bi(2,3)–Pb5 3.11, Au1···Au1^{i} 2.92, Au1···Pb(1,2) 3.16, Au1–Pb(1^{i},2^{i}) 2.96, Au1–Bi(2,3) 2.87.

5.4 Bonding situation of the Au atoms

The results of the Paboon analysis are provided in Table S10.

Table S10. Calculated shared electron (SEN) numbers for the Au···Au contacts in the anions {[AuTt$_2$Pn$_2$]$_2$}$^{2-}$ (Tt: Sn, Pb; Pn: Sb, Bi) and averaged SEN numbers for the Tt–Tt bonds for comparison.

Anion	Bond	SEN
$[(AuSn_2Sb_2)_2]^{2-}$	Au···Au	0.1152
	Ø(Sn–Sn)	0.8871
$[(AuSn_2Bi_2)_2]^{2-}$	Au···Au	0.1246
	Ø(Sn–Sn)	0.9249
$[(AuPb_2Sb_2)_2]^{2-}$	Au···Au	0.1579
	Ø(Pb–Pb)	0.7661
$[(AuPb_2Bi_2)_2]^{2-}$	Au···Au	0.1416
	Ø(Pb–Pb)	0.8045

Figures S33–S35 show the localized molecular orbitals (LMOs) of {[AuPb$_5$Sb$_2$]$_2$}$^{4-}$ with largest contributions from the Au atoms, mainly involved in three-center bonds with two main group atoms. These LMOs look qualitatively the same for all four isostructural anions {[AuTt$_5$Pn$_3$]$_2$}$^{4-}$ studied herein. Additional aurophilic interactions between the two Au atoms may be present, but the fact that the two subunits hold together, obviously may be rationalized without them.

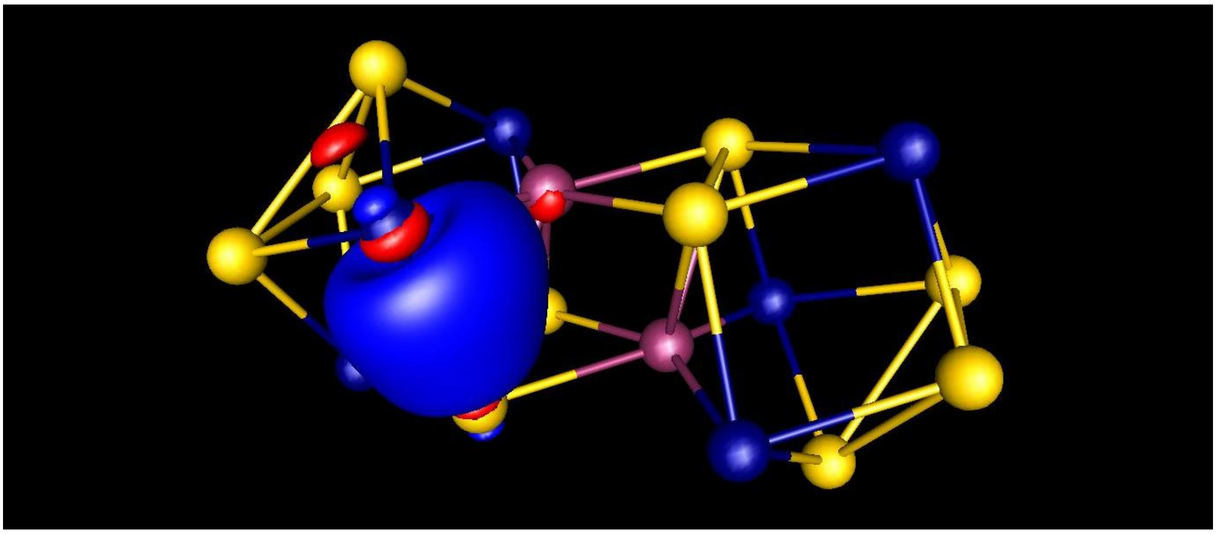

Figure S33. Localized molecular orbital of the 3-center interaction between the atoms Pb1, Bi2, and Au1 (Pb: yellow, Pb: blue, Au: purple; contours drawn at ±0.048 a.u.).

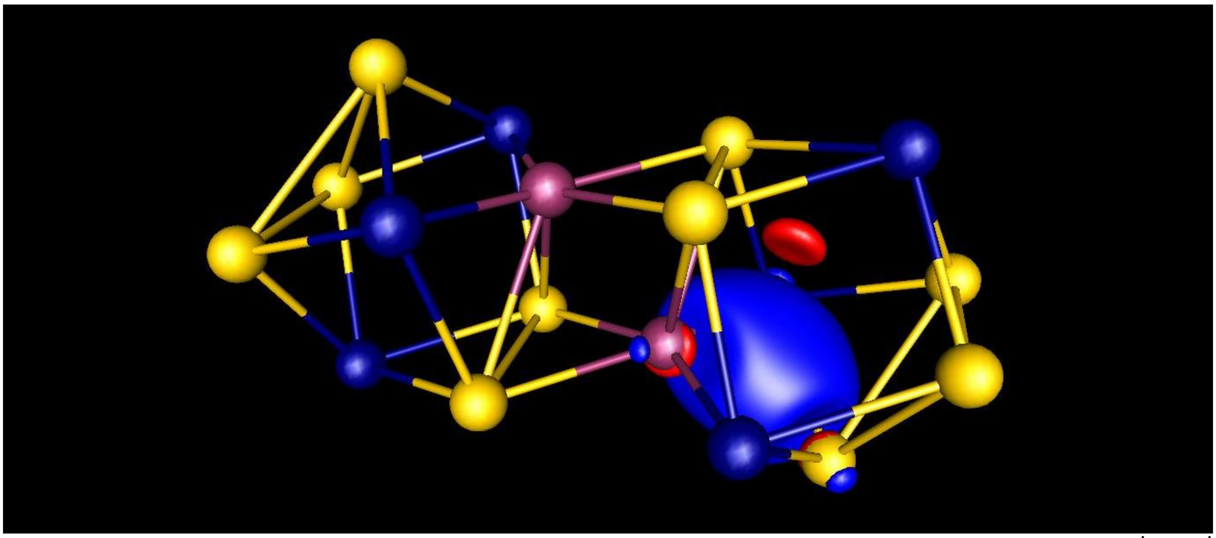

Figure S34. Localized molecular orbital of the 3-center interaction between the atoms Pb5^{i}, Bi2^{i}, and Au1^{i} (Pb: yellow, Pb: blue, Au: purple; contours drawn at ±0.048 a.u.).

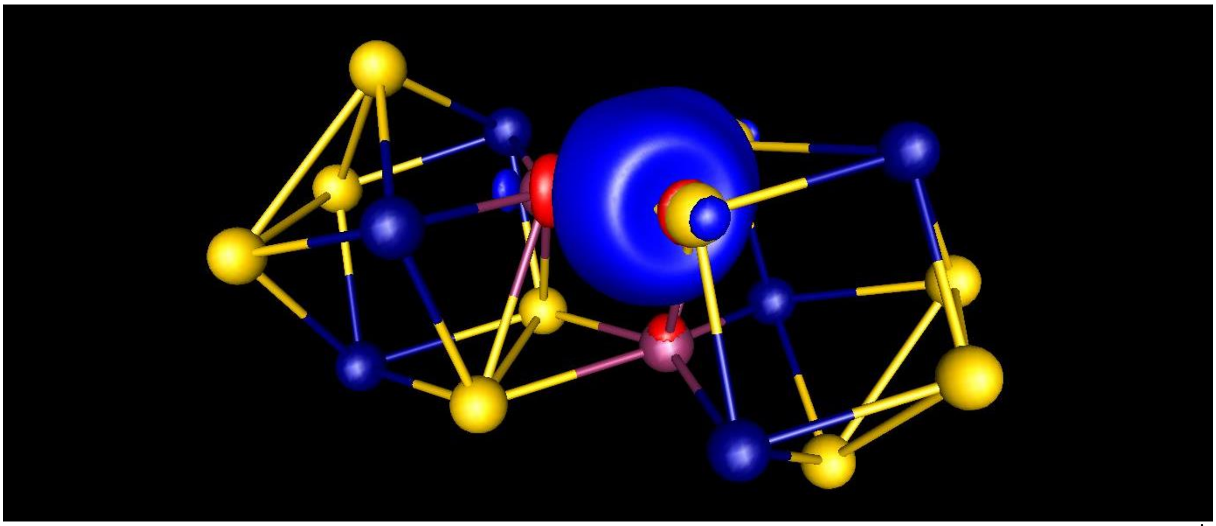

Figure S35. Localized molecular orbital of the 3-center interaction between the atoms Pb1^{i}, Pb2^{i}, and Au1 (Pb: yellow, Pb: blue, Au: purple; contours drawn at ±0.048 a.u.).

6. References for the Supporting Information

[1] 4,7,13,16,21,24-Hexaoxa-1,10-diazabicyclo[8.8.8]hexacosane.

[2] a) S. Mitzinger, L. Broeckaert, W. Massa, F. Weigend, S. Dehnen, *Nat. Commun.* **2016**, *7*, 10480–10490; b) S. C. Critchlow, J. D. Corbett, *Inorg. Chem.* **1982**, *21*, 3286–3290; c) F. Lips, I. Schellenberg, R. Pöttgen, S. Dehnen, *Chem. Eur. J.* **2009**, *15*, 12968–12973; d) R. Ababei, J. Heine, M. Hołyńska, G. Thiele, B. Weinert, X. Xie, F. Weigend, S. Dehnen, *Chem. Commun.* **2012**, *48*, 11295–11297.

[3] Sheldrick, G. M. *Acta Crystallographica Section A: Foundations and Advances* **2015**, *71*, 3.

[4] Sheldrick, G.M. *Acta Crystallographica Section C: Structural Chemistry* **2015**, *71*, 3.

[5] Diamond - Crystal and Molecular Structure Visualization, Crystal Impact - Dr. H. Putz & Dr. K. Brandenburg GbR, Kreuzherrenstr. 102, 53227 Bonn, Germany, http://www.crystalimpact.com/diamond.

[6] TURBOMOLE V7.4.1 2019, a development of University of Karlsruhe and Forschungszentrum Karlsruhe GmbH, 1989–2007, TURBOMOLE GmbH, since 2007; available from http://www.turbomole.com.

[7] S. G. Balasubramani, G. P. Chen, S. Coriani, M. Diedenhofen, M. S. Frank, Y. J. Franzke, F. Furche, R. Grotjahn, M. E. Harding, C. Hättig, A. Hellweg, B. Helmich-Paris, C. Holzer, U. Huniar, M. Kaupp, A. M. Khah, S. K. Khani, T. Müller, F. Mack, B. D. Nguyen, S. M. Parker, E. Perlt, D. Rappoport, K. Reiter, S. Roy, M. Rückert, G. Schmitz, M. Sierka, E. Tapavicza, D. P. Tew, C. van Wüllen, V. K. Voora, F. Weigend, A. Wodyński, and J. M. Yu. J. Chem. Phys. 2020, 152, 184107.

[8] J. Tao, J. P. Perdew, V. N. Staroverov, G. E. Scuseria, *Phys. Rev. Lett.* **2003**, *91*, 3–6.

[9] a) F. Weigend, R. Ahlrichs, *Phys. Chem. Chem. Phys.* **2005**, *7*, 3297–3305; b) F. Weigend, A. Baldes, *J. Chem. Phys.* **2010**, *133*, 174102.

[10] F. Weigend, *Phys. Chem. Chem. Phys.* **2006**, *8*, 1057–1065.

[11] a) B. Metz, H. Stoll, M. Dolg, *J. Chem. Phys.* **2000**, *113*, 2563–2569; b) D. Figgen, G. Rauhaut, M. Dolg, H. Stoll, *Chem. Phys.* **2005**, *311*, 227–244.

[12] C. Ehrhardt, R. Ahlrichs, *Theor. Chim. Acta* **1985**, *68*, 231–245.

[13] S. F. Boys, in *Quantum Theory of Atoms, Molecules and the Solid State* (Ed.: P.-O. Löwdin), New York, **1966**, 253–262.

[14] a) L. Laaksonen, *J. Mol. Graph.* **1992**, *10*, 33–34; b) D. L. Bergman, L. Laaksonen, A. Laaksonen, *J. Mol. Graph. Model.* **1997**, *15*, 301–306.

[15] B. Cordero, V. Gómez, A. E. Platero-Prats, M. Revés, J. Echevarría, E. Cremadés, F. Barragán, S. Alvarez, *Dalton Trans.* **2008**, *21*, 2832–2838.
